# Supplementary material for: Structure, catalysis, chitin transport, and selective inhibition of chitin synthase
Source: Nat Commun. 2023 Aug 8;14:4776. doi: 10.1038/s41467-023-40479-4 (PMC10409773; doi:10.1038/s41467-023-40479-4)
Supplement: Supplementary file 1 — Supplementary Information [file 41467_2023_40479_MOESM1_ESM.pdf]

Supplementary information

Supplementary Table 1. Cryo-EM data collection, refinement, and validation.

Sample	*S. cerevisiae* Chs1							
Incubated with	-	*GlcNAc*	*UDP-GlcNAc 5min*	*UDP-GlcNAc 40 min*	*UDP-GlcNAc +GlcNAc*	*UDP*	*PolyB*	*NikkoZ*
State	***Apo***	***Apo***	*UDP-GlcNAc*	*UDP+ GlcNAc*	*UDP-GlcNAc +GlcNAc*	*UDP*	*PolyB*	*NikkoZ*
Map and Model	**EMD-36856, PDB ID: 8K3Q**	**EMD-36857**	**EMD-36862, PDB ID: 8K3V**	**EMD-36861, PDB ID: 8K3U**	**EMD-36863, PDB ID: 8K3W**	**EMD-36859, PDB ID: 8K3T**	**EMD-36855, PDB ID: 8K3P**	**EMD-36864, PDB ID: 8K3X**
**Data collection and processing**								
Microscope	FEI Titan Krios							
Voltage (kV)	300							
Electron exposure (e^{-}/Å^2)	50							
Defocus range (-μm)	1.0-2.0							
Pixel size (Å)	1.06	1.06	1.06	1.06	1.06	1.06	1.06	1.06
Micrographs (no.)	1914	1564	3045	2469	1356	632	2311	2711
Initial particle images (no.)	2,704,516	1,077,654	3,784 , 484	1,059,472	1,920,416	431,546	2,508,153	4,135,734
Final particle images (no.)	416,536	218,698	250,346	184,449	107,259	115,773	142,887	837,015
Symmetry imposed	C2	C2	C2	C2	C2	C2	C2	C2
Map resolution (Å)<br>FSC threshold	2.60<br>0.143	3.51<br>0.143	3.10<br>0.143	3.06<br>0.143	2.91<br>0.143	3.57<br>0.143	3.64<br>0.143	2.44<br>0.143
Map resolution range (Å)	250 - 2.60	250 - 3.51	250 - 3.10	250 - 3.06	250 - 2.91	250 - 3.57	250 - 3.64	250 - 2.44
**Refinement**								
Map sharpening *B* factor (Å^2)	102.5	161.9	120.1	129.1	91.8	151.2	155.5	107.4
Model composition<br>Nonhydrogen atoms<br>Protein residues	12340<br>1504	-	11862<br>1438	11070<br>1334	11892<br>1438	11834<br>1438	11852<br>1438	11838<br>1436
R.m.s. deviations								

Bond lengths (Å)<br>Bond angles (°)	0.01<br>0.83	-	0.01<br>0.70	0.01<br>0.74	0.01<br>0.66	0.01<br>0.86	0.01<br>0.77	0.01<br>0.91
Validation								
MolProbity score<br>Clash score<br>Poor rotamers (%)<br>FSC(model-map) =0.5	1.85<br>8.4<br>0.2<br>2.9	-	1.93<br>8.5<br>0.2<br>3.3	2.00<br>16.7<br>0.0<br>3.7	1.75<br>8.15<br>0.2<br>3.2	2.50<br>16.0<br>0.2<br>3.9	2.30<br>12.9<br>0.2<br>3.9	1.80<br>7.6<br>0.2<br>2.8
Ramachandran plot								
Favored (%)<br>Allowed (%)<br>Disallowed (%)	96.22<br>3.78<br>0	-	95.42<br>4.58<br>0	96.04<br>3.96<br>0	95.56<br>4.44<br>0	95.42<br>4.58<br>0	94.78<br>5.22<br>0	94.42<br>5.58<br>0

Supplementary Table 2. Primer sequences for generating Chs1 FLAG tagging and mutations.

	Forward primer (5'—>3')	Reverse primer (5'—>3')
FLAG-tag	GAATTCGATATCAAGCTTATCATGAGTGATCAAAATAATCGATCGAGAAATG	GACTCGAGGTCGACGGTATCGCGAAATGTAACCTTTTTAAAAAACCTTACTATCATG
Chs1Δ	CGAAGAGTCACTAAATTAATACTGGGAAGCCAAACCAAAAAAACTATACAGACATGGAGGCCCAGAATACCCT	ATGATAGTAAACACATATACTTATTTATAACAAAGGGGTAATCAATCTACCAGTATAGCGACCAGCATTCACATAC
ΔN	GGAATTCGATATCAAGCATGGGTGTACCTGCATCATCCTCGGTGTC	CATGCTTGATATCGAATTCCTGCAGC
N456A/E457A	GCTGCTGACCATATCCTGTTAGGAAGAACTTTGAAAGG	CTAACAGGATATGGTCAGCAGCATACATTGTGACTACTAGCATCAATTCCGT
K578A	AGAGCAAAATCAGAAAGCTATTAACTCACATAGATGGGCATTTG	AGCTTTCTGATTTTGCTCTTTCAAACAAAACAAAAGTTG
D602A	ATATCGTTACATTGTTAGCTGCTGGTACCATGCCAG	AGCTAACAATGTAACGATATTGGGACGCA
S657N/N658F	TTCGAATACAAAATGTTTTTCATTTTAGACAAAACAACCGAGTCTAACTTTG	TGTTTTGTCTAAAATGAAAAACATTTTGTATTCGAAATTCTGTGATGCAAC
K662M	ATGTCCAATATTTTAGACATGACAACCGAGTCTAACTTTGGATTTATTACTG	AAAGTTAGACTCGGTTGTCATGTCTAAAATATTGGACATTTTGTATTCG
V673S	AACTTTGGATTTATTACTTCTCTACCGGGGGCATTCTCTGC	AGAGAATGCCCCCGGTAGAGAAGTAATAAATCCAAAGTTAGACTCGGTTG
L674Q	TTTGGATTTATTACTGTTCAGCCGGGGGCATTCTCTGCGTATAG	CGCAGAGAATGCCCCCGGCTGAACAGTAATAAATCCAAAGTTAGACTCGG
P675L	GGATTTATTACTGTTCTATTGGGGGCATTCTCTGCGTATAGGTTTG	ATACGCAGAGAATGCCCCCAATAGAACAGTAATAAATCCAAAGTTAGACTCGG
P675T	GGATTTATTACTGTTCTAACAGGGGCATTCTCTGCGTATAGGTTTG	ATACGCAGAGAATGCCCCTGTTAGAACAGTAATAAATCCAAAGTTAGACTCGG
G676L	TTTATTACTGTTCTACCGTTGGCATTCTCTGCGTATAGGTTTGAAGCTG	CCTATACGCAGAGAATGCCAACGGTAGAACAGTAATAAATCCAAAGTTAGAC
R718L	ATGTATCTTGCTGAAGATTTGATTTTATGCTTTGAAGTGGTCAC	CACTTCAAAGCATAAAATCAAATCTTCAGCAAGATACATATTGGAAG
D745R	AGTTCTTATGCTTCAACAAGAGT	AGGGACCCTCTCCGGTACTCTTGT

	ACCGGAGAGGGTCCCTGAATTTATTC	TGAAGCATAAGAACTTCTGCAGTATTTC
E752R	GTACCGGAGAGGGTCCCTAGATTTATTCTTCAGAGGAGGCGTTGGTTGA	CCTCCTCTGAAGAATAAATCTAGGGACCCTCTCCGGTACATCTG
Q756L	GTCCCTGAATTTATTCTTTTGAGGAGGCGTTGGTTGAATGGTTC	ATTCAACCAACGCCTCCTCAAAAGAATAAATTCAGGGACCCTCTCCGG
R759A	GCTTGGTTGAATGGTTCATTTTTTGCTAG	AATGAACCATTCAACCAAGCCCTCCTCTGAAGAATAAATTCAGG
W760A	ATTCTTCAGAGGAGGCGTGCTTTGAATGGTTCATTTTTTGCTAGTGTATATTC	AAAAAATGAACCATTCAAAGCACGCCTCCTCTGAAGAATAAATTCAGG
W969A	TAATGTCCACGACTTATCAGCTGGTACAAAGGGTGCAATGGCAAATC	AGCTGATAAGTCGTGGACATTACAAAATGCATAG
G970A	TAATGTCCACGACTTATCAGCTACAAAGGGTGCAATGGCAAATCC	AGCCCATGATAAGTCGTGGACATTACAAAATGC
S883F	TTTATATTCATGAGTGTCAAATCCTTCCAAAATATATTG	TTGACACTCATGAATATAAAGCAGAATATCATGTAAATCATCATCACCGCA
S917F	TTTCTGGGCTCCACTTATTGTTTGTACC	AATAAGTGGAGCCCAGAAAGATAACAATATCCCTGAAAGCTTCTGTGGT
ΔSL	GAGCCATCTTATGATGAAAAAAAGACTGGC	TTTTCATCATAAGATGGCTCTGCACCCTTTGTACCCCATGATAAG

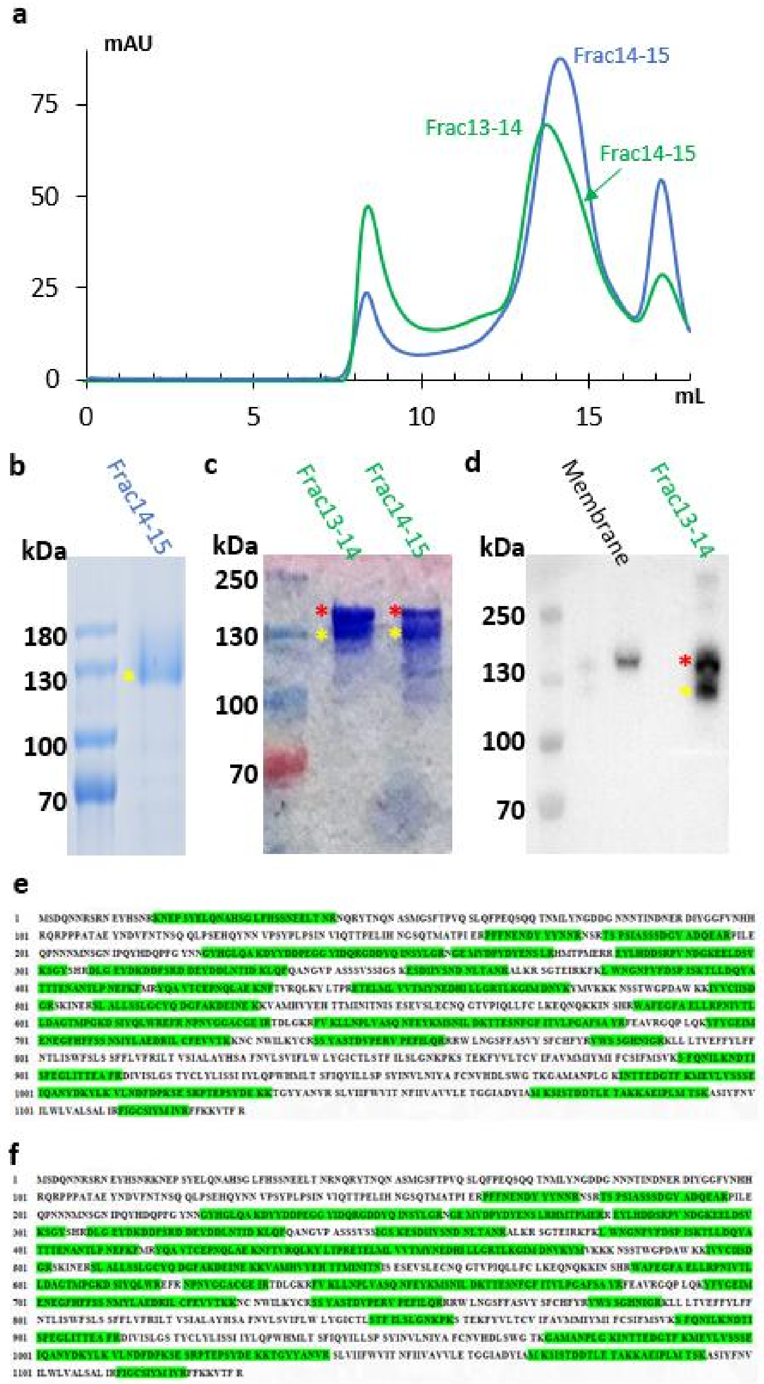

Supplementary Figure 1. Purification and analysis of Chs1. **a**) Gel filtration profile of Chs1. Sample purified with regular procedure was shown in blue, while sample purified with excess protease inhibitor in all buffers during purification was shown in green. The peak fractions were checked by Coomassie blue-stained SDS–PAGE gel (**b-c**). Two major bands locating higher or lower than 130kDa marker are highlighted by red or yellow star respectively. **d**) Western blot analysis of the cell membrane or peak fraction in (**a**). Source data of panel **b-d** are provided as a Source Data file. Three independent experiments of **b-d** were conducted with similar results. **e-f**) Tryptic digestion mass spectrometry for two bands in (**c**). The detected peptides are highlighted in green. Source data is provided as a Source Data file.

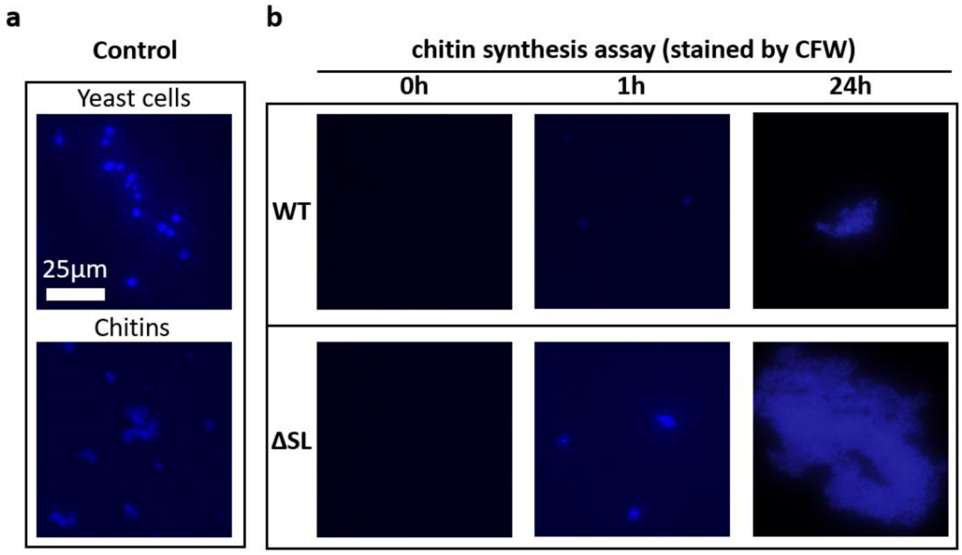

Supplementary Figure 2. Characterization of synthesized chitin by CFW staining. **a**) As references, cell walls of yeast cells and commercial chitins were stained using CFW. **b**) In vitro chitin synthesis assay of purified Chs1 (WT) and a swapping loop truncated Chs1 (Δ SL) at different time points (0, 1, 24 hours) by staining the product using CFW. Three independent experiments of **a-b** were conducted with similar results.

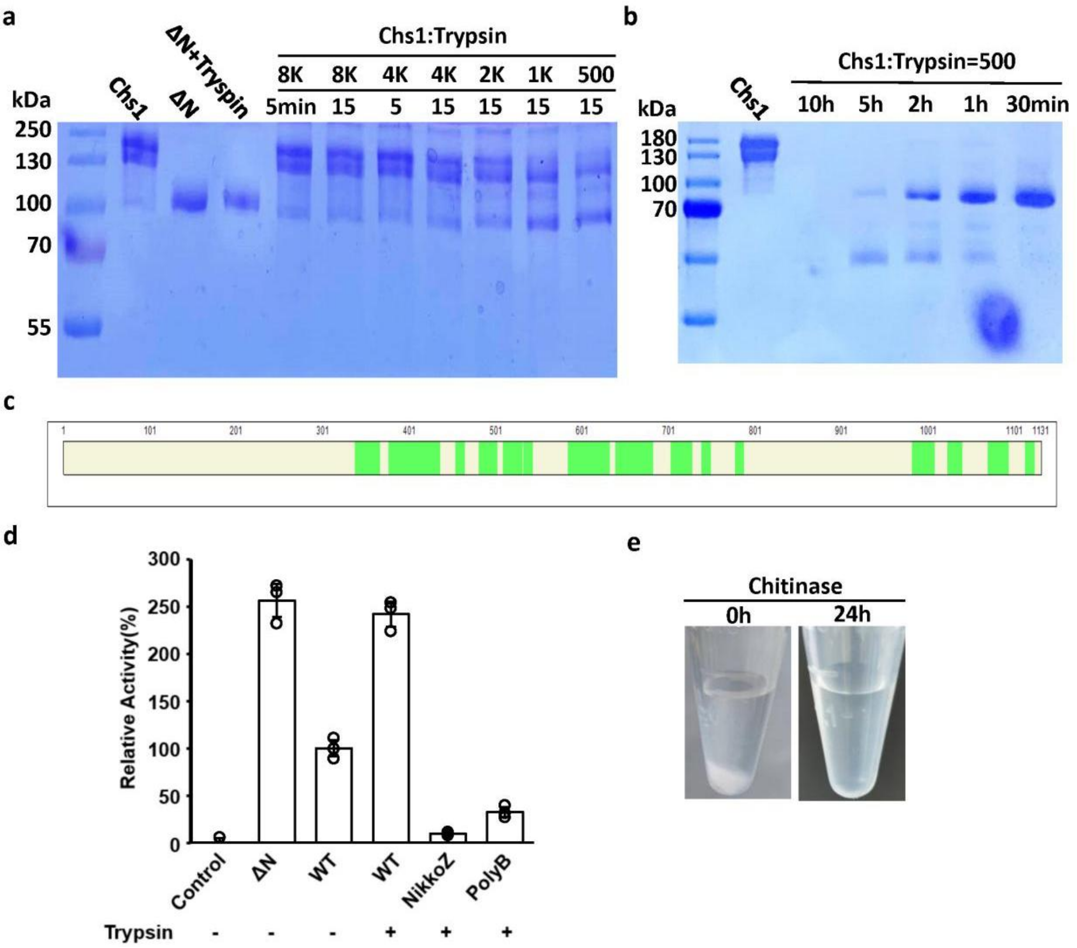

Supplementary Figure 3. Proteolysis analysis of Chs1 by trypsin. a) Purified Chs1 and a truncated Chs1 by deleting N-terminal 340 residues (ΔN) were incubated with trypsin in different mass ratios (500:1, 1000:1, 2000:1, 4000:1, 8000:1) and times (5mins, 15mins), and then checked by Coomassie blue-stained SDS–PAGE gel. Source data is provided as a Source Data file. Three independent experiments were conducted with similar results. **b**) Equal amounts of purified Chs1 were first incubated with trypsin in mass ratios at 500:1 for different times (30mins, 1 hour, 2 hours, 5 hours or 10 hours), and then checked by Coomassie blue-stained SDS–PAGE gel. Source data is provided as a Source Data file. Three independent experiments were conducted with similar results. **c**) Tryptic digestion mass spectrometry for the proteolytic band in (**b**). The detected peptides are highlighted in green. Source data is provided as a Source Data file. **d**) In vitro UDP-Glo glycosyltransferase assay with wild type Chs1 and ΔN in buffer with or without trypsin, NikkoZ, or PolyB. The reaction without Chs1 was used as the control. Data points represent the mean ± SD in triplicate. **e**) Chitinase digestion of synthesized product by trypsin activated Chs1 (24h reaction). The synthesized chitin was visible as white precipitant and disappeared after 24h incubation with chitinase. Three independent experiments were conducted with similar results.

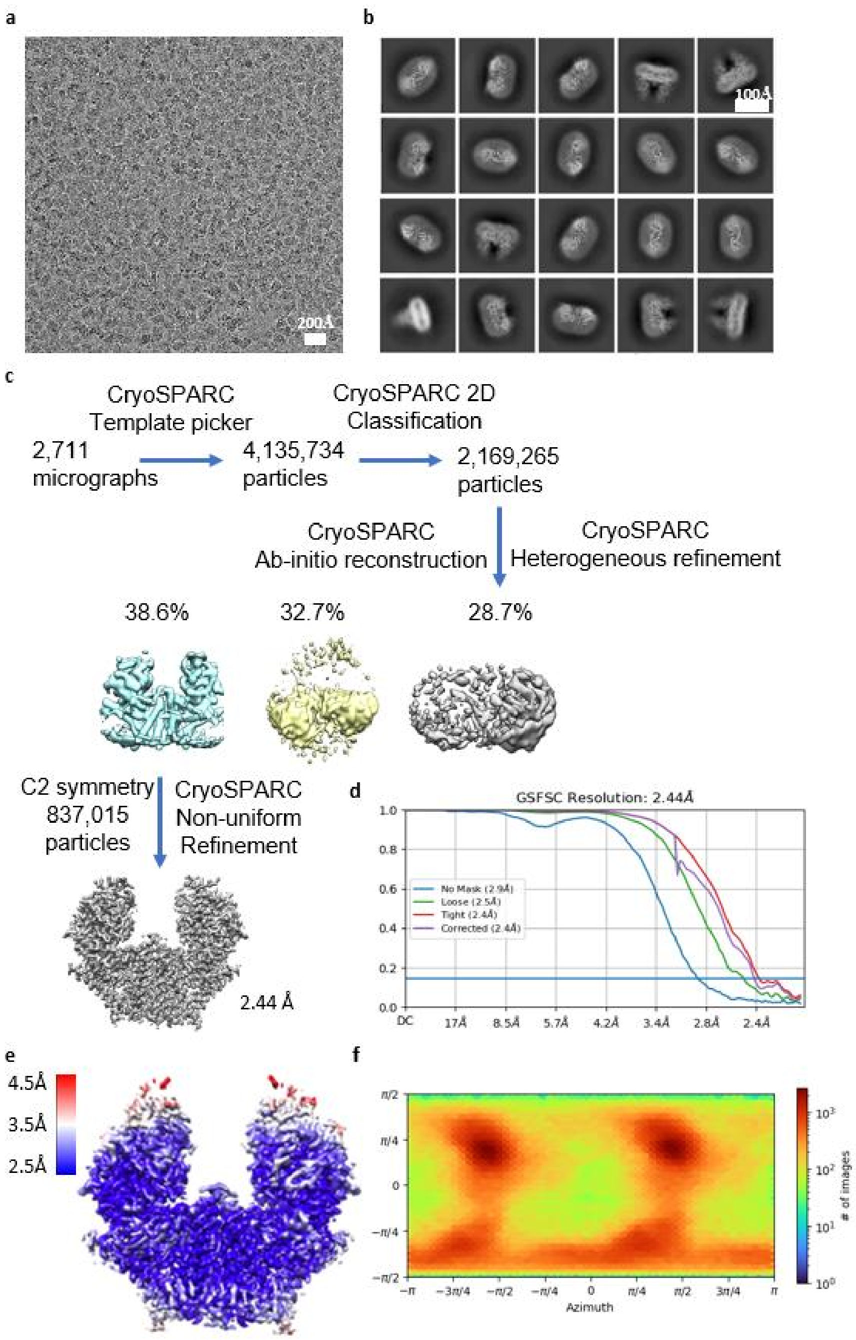

Supplementary Figure 4. Cryo-EM analysis of Chs1 in complex with NikkoZ. a) A representative electron micrograph. **b**) Selected reference-free 2D class averages. **c**) Cryo-EM data processing procedure. **d**) Gold-standard Fourier shell correlation of two independent half 3D maps of Chs1 in C2 symmetry. **e**) Local resolution map of the 2.44-Å 3D map of NikkoZ-bound Chs1 in C2 symmetry. **f**) Angular distribution of raw particles used in CryoSPARC 3D reconstruction of the NikkoZ-bound Chs1.

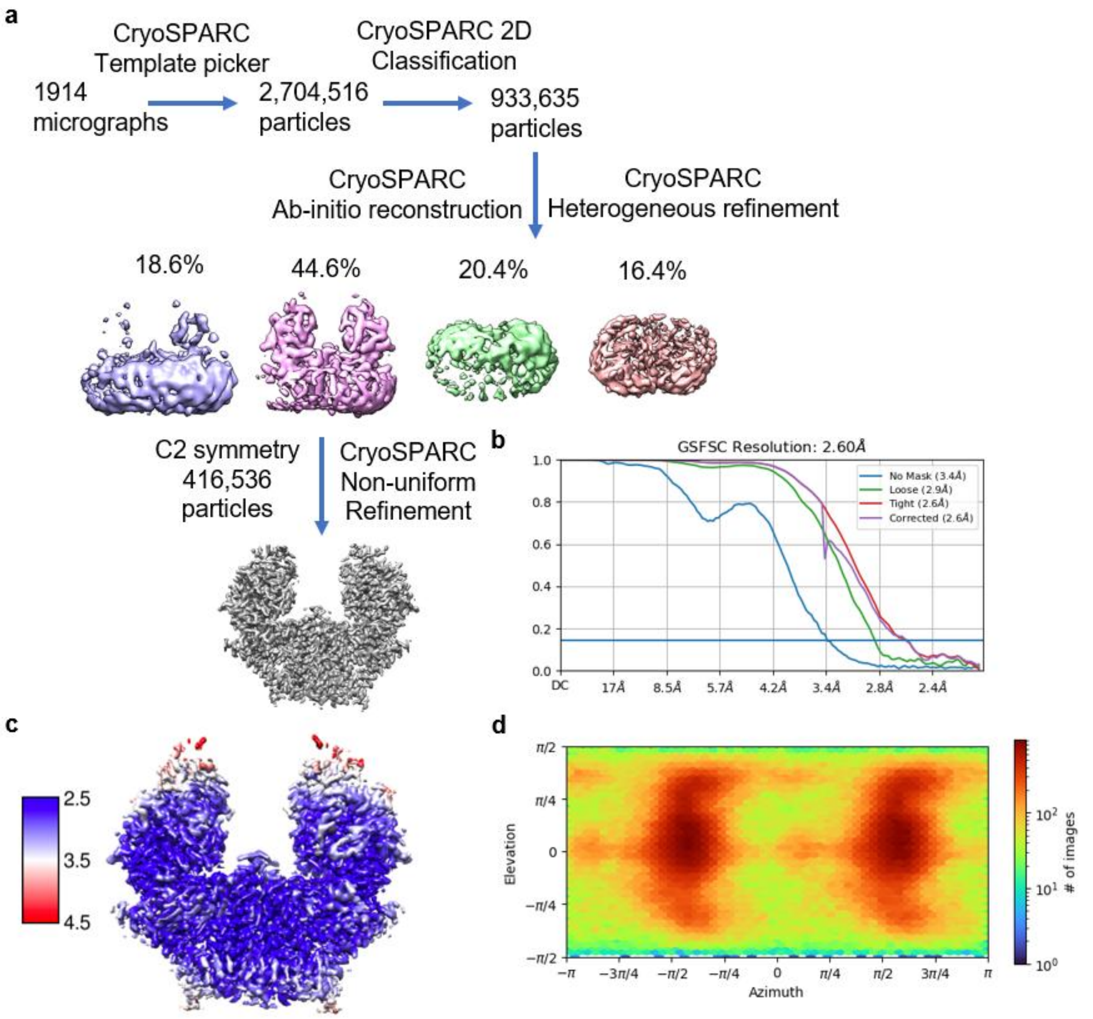

Supplementary Figure 5. Cryo-EM data processing and resolution estimation of Chs1 in the apo state. **a**) Cryo-EM data processing procedure. **b**) Gold-standard Fourier shell correlation of two independent half 3D maps of Chs1 in C2 symmetry. **c**) Local resolution map of the 2.60-Å 3D map of apo Chs1 in C2 symmetry. **d**) Angular distribution of raw particles used in CryoSPARC 3D reconstruction of the apo Chs1.

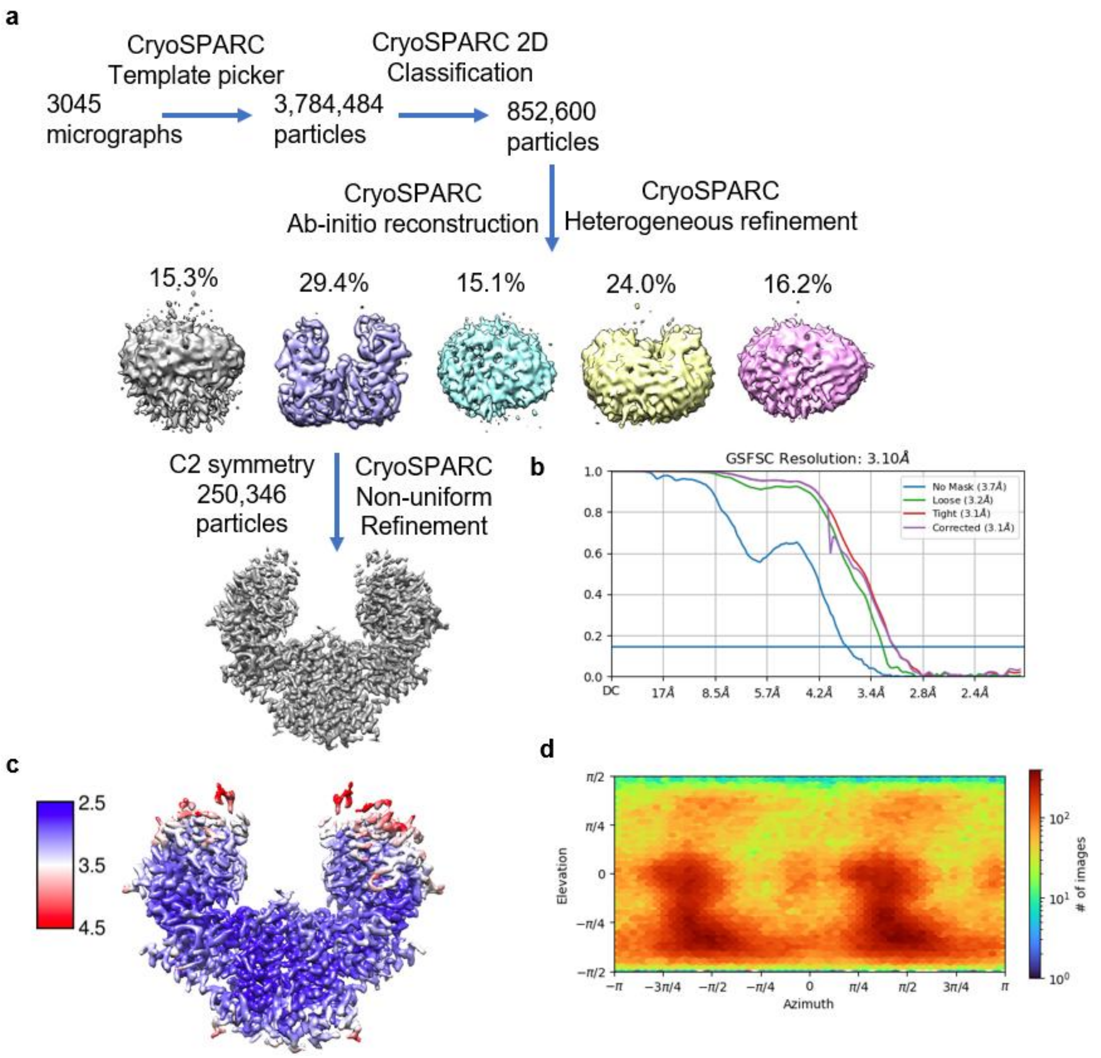

Supplementary Figure 6. Cryo-EM data processing and resolution estimation of UDP-GlcNAc+GlcNAc bound Chs1. a) Cryo-EM data processing procedure. **b**) Gold-standard Fourier shell correlation of two independent half 3D maps of Chs1 in C2 symmetry. **c**) Local resolution map of the 3.10-Å 3D map of UDP-GlcNAc+GlcNAc bound Chs1 in C2 symmetry. **d**) Angular distribution of raw particles used in CryoSPARC 3D reconstruction of UDP-GlcNAc+GlcNAc bound Chs1.

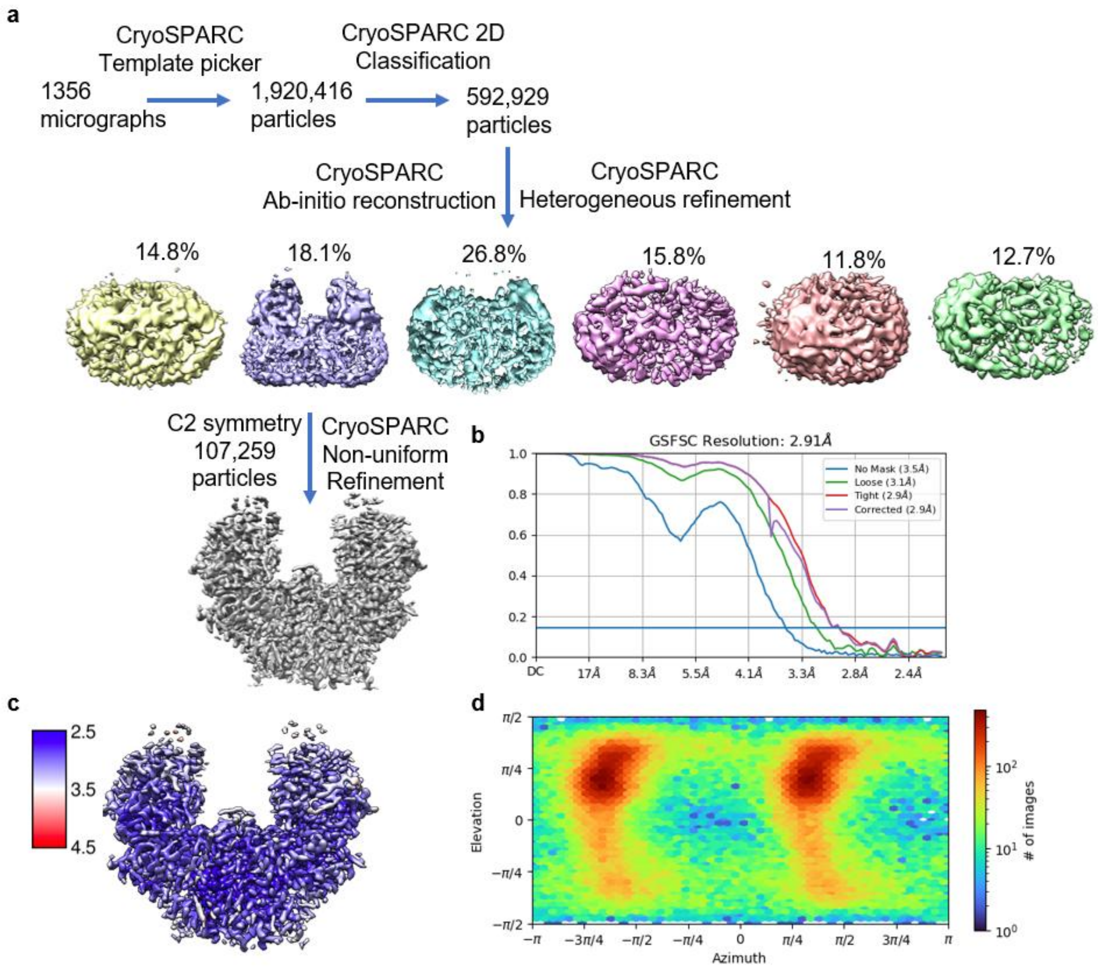

Supplementary Figure 7. Cryo-EM data processing and resolution estimation of UDP-GlcNAc bound Chs1. **a**) Cryo-EM data processing procedure. **b**) Gold-standard Fourier shell correlation of two independent half 3D maps of Chs1 in C2 symmetry. **c**) Local resolution map of the 2.91-Å 3D map of UDP-GlcNAc bound Chs1 in C2 symmetry. **d**) Angular distribution of raw particles used in CryoSPARC 3D reconstruction of UDP-GlcNAc-bound Chs1.

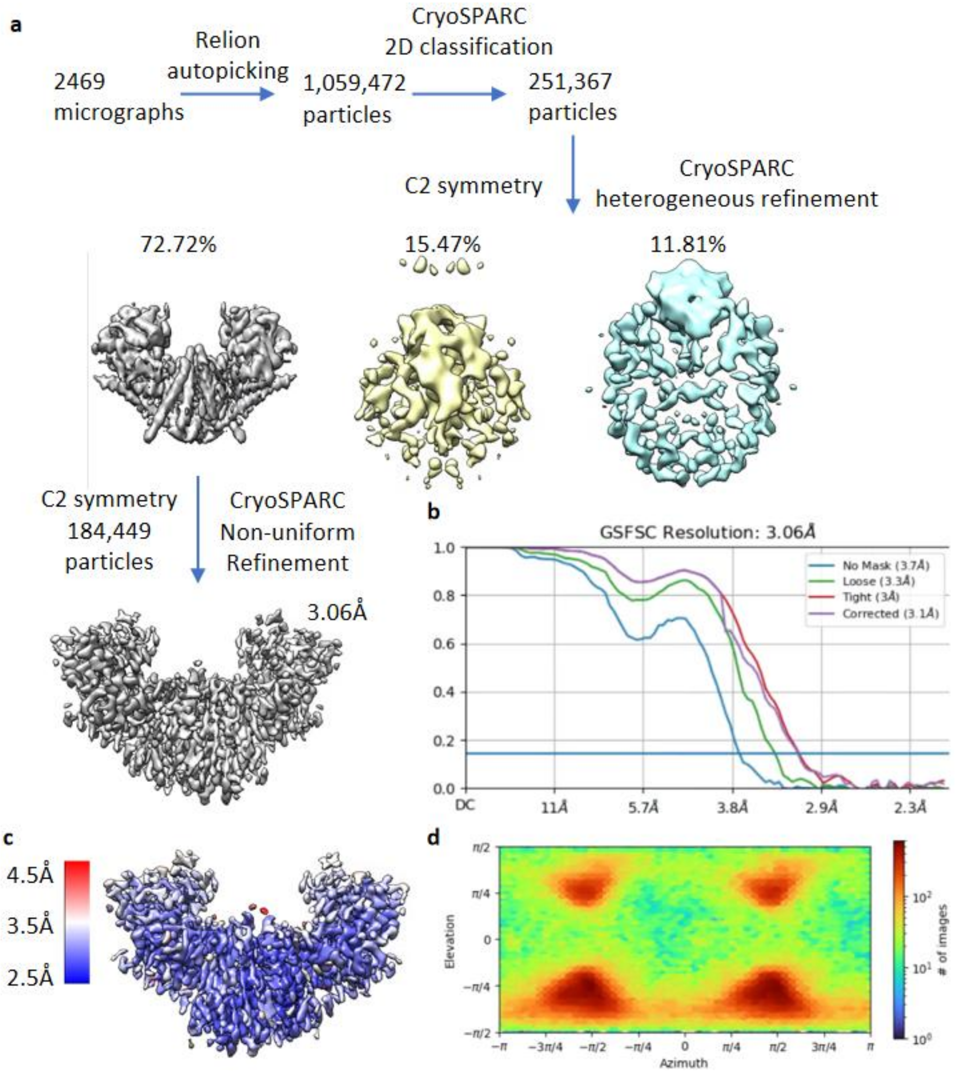

Supplementary Figure 8. Cryo-EM data processing and resolution estimation of primed Chs1 (UDP+GlcNAc bound). **a**) Cryo-EM data processing procedure. **b**) Gold-standard Fourier shell correlation of two independent half 3D maps of Chs1 in C2 symmetry. **c**) Local resolution map of the 3.06-Å 3D map of primed Chs1 in C2 symmetry. **d**) Angular distribution of raw particles used in CryoSPARC 3D reconstruction of the primed Chs1.

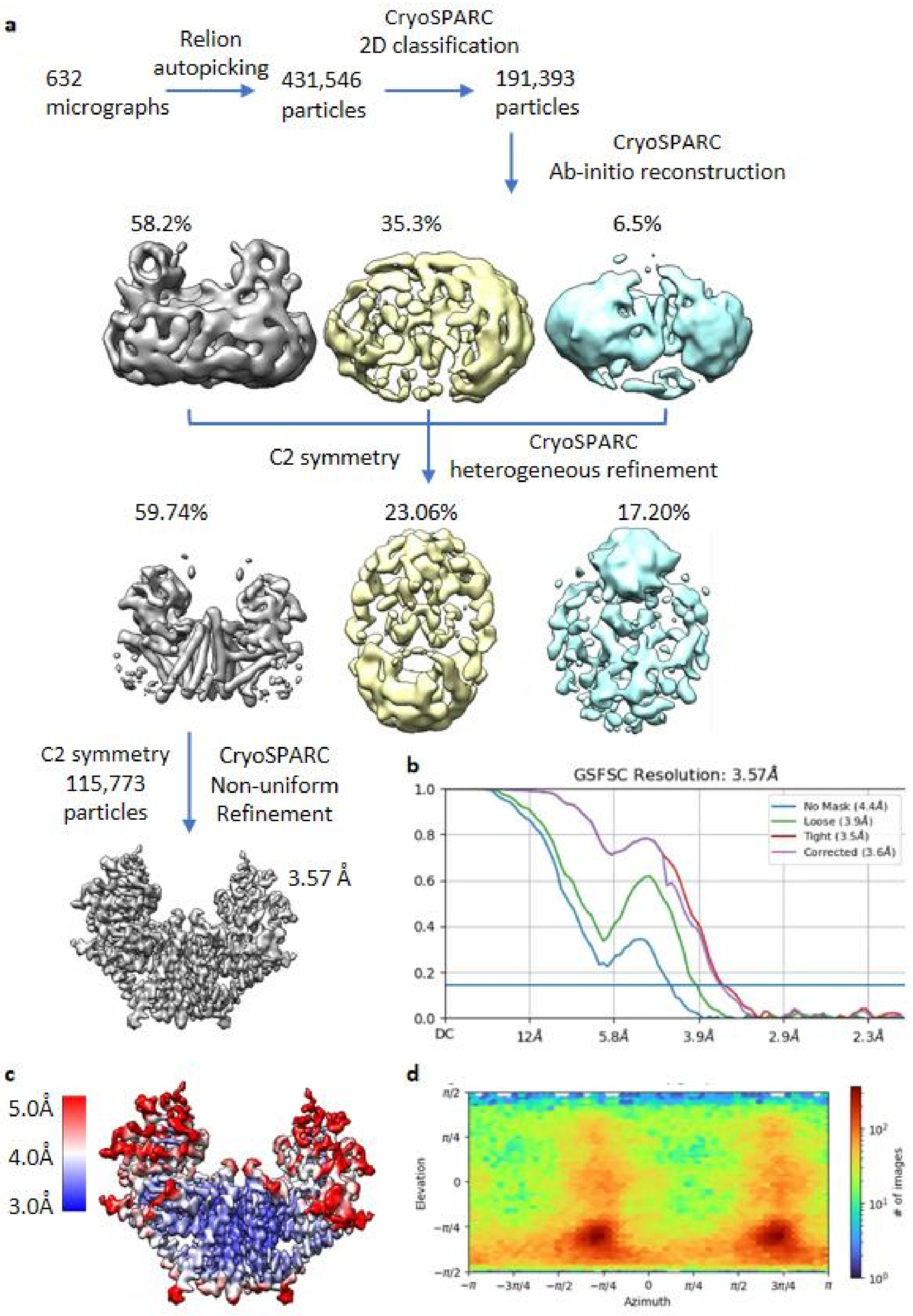

Supplementary Figure 9. Cryo-EM data processing and resolution estimation of UDP-bound Chs1. a) Cryo-EM data processing procedure. **b**) Gold-standard Fourier shell correlation of two independent half 3D maps of Chs1 in C2 symmetry. **c**) Local resolution map of the 3.57-Å 3D map of UDP-bound Chs1 in C2 symmetry. **d**) Angular distribution of raw particles used in CryoSPARC 3D reconstruction of UDP-bound Chs1.

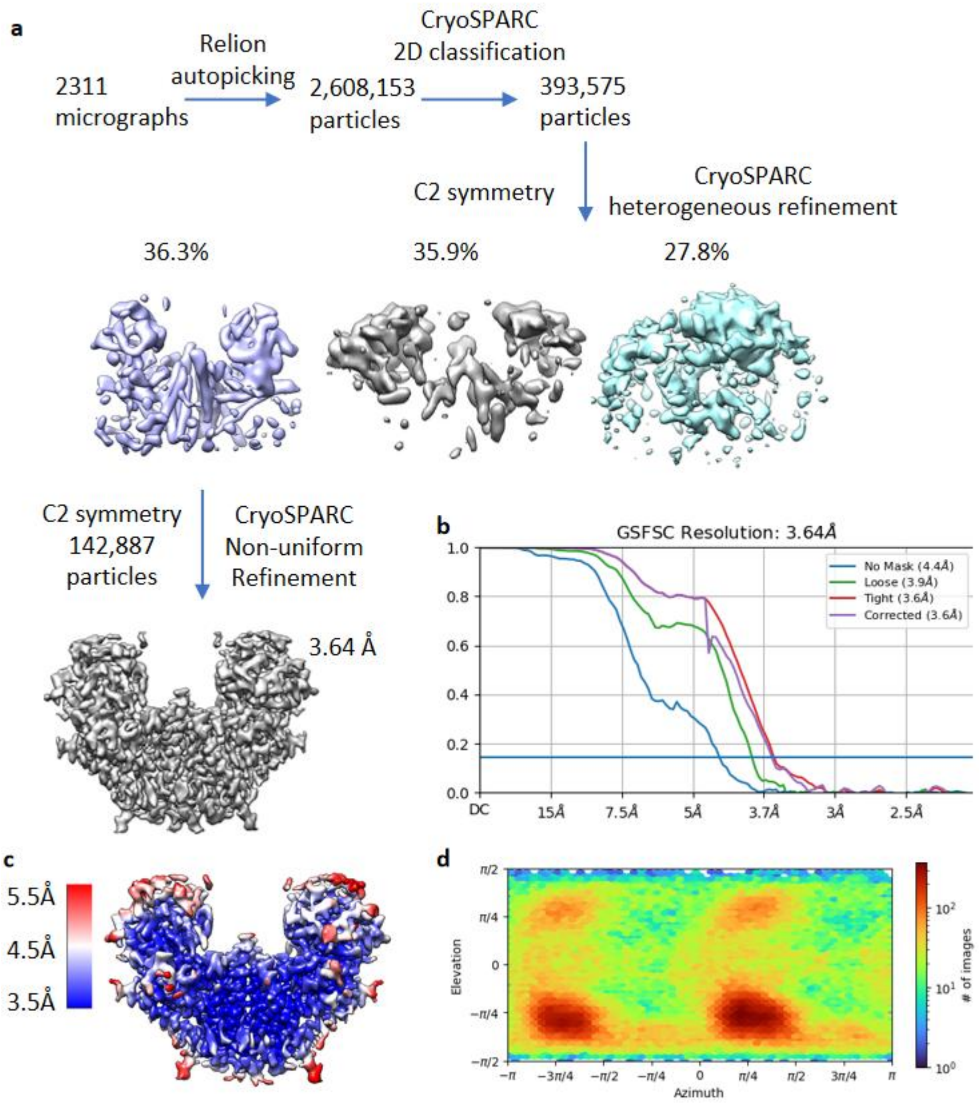

Supplementary Figure 10. Cryo-EM data processing and resolution estimation of PolyB bound Chs1. **a**) Cryo-EM data processing procedure. **b**) Gold-standard Fourier shell correlation of two independent half 3D maps of Chs1 in C2 symmetry. **c**) Local resolution map of the 3.64-Å 3D map of PolyB-bound Chs1 in C2 symmetry. **d**) Angular distribution of raw particles used in CryoSPARC 3D reconstruction of PolyB-bound Chs1.

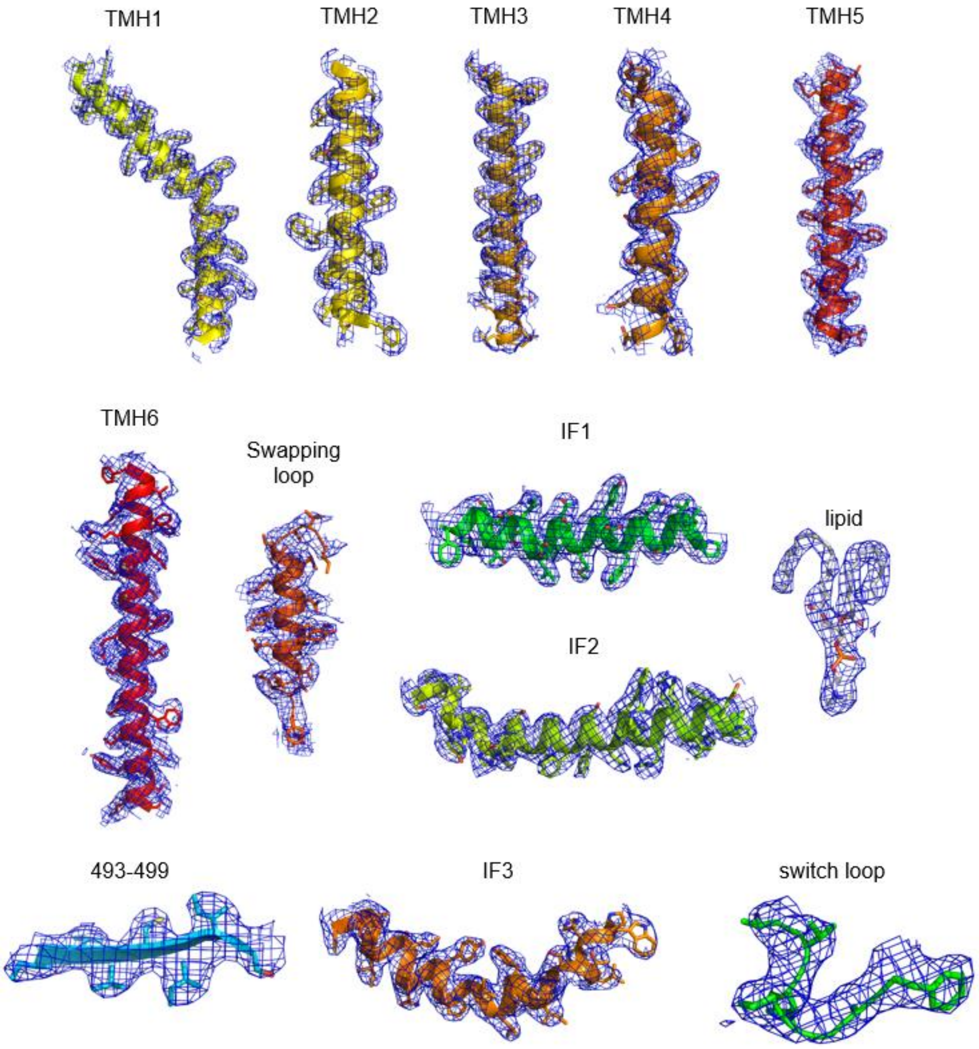

Supplementary Figure 11. Selected regions in the 3D map of apo Chs1 superimposed with the atomic model.

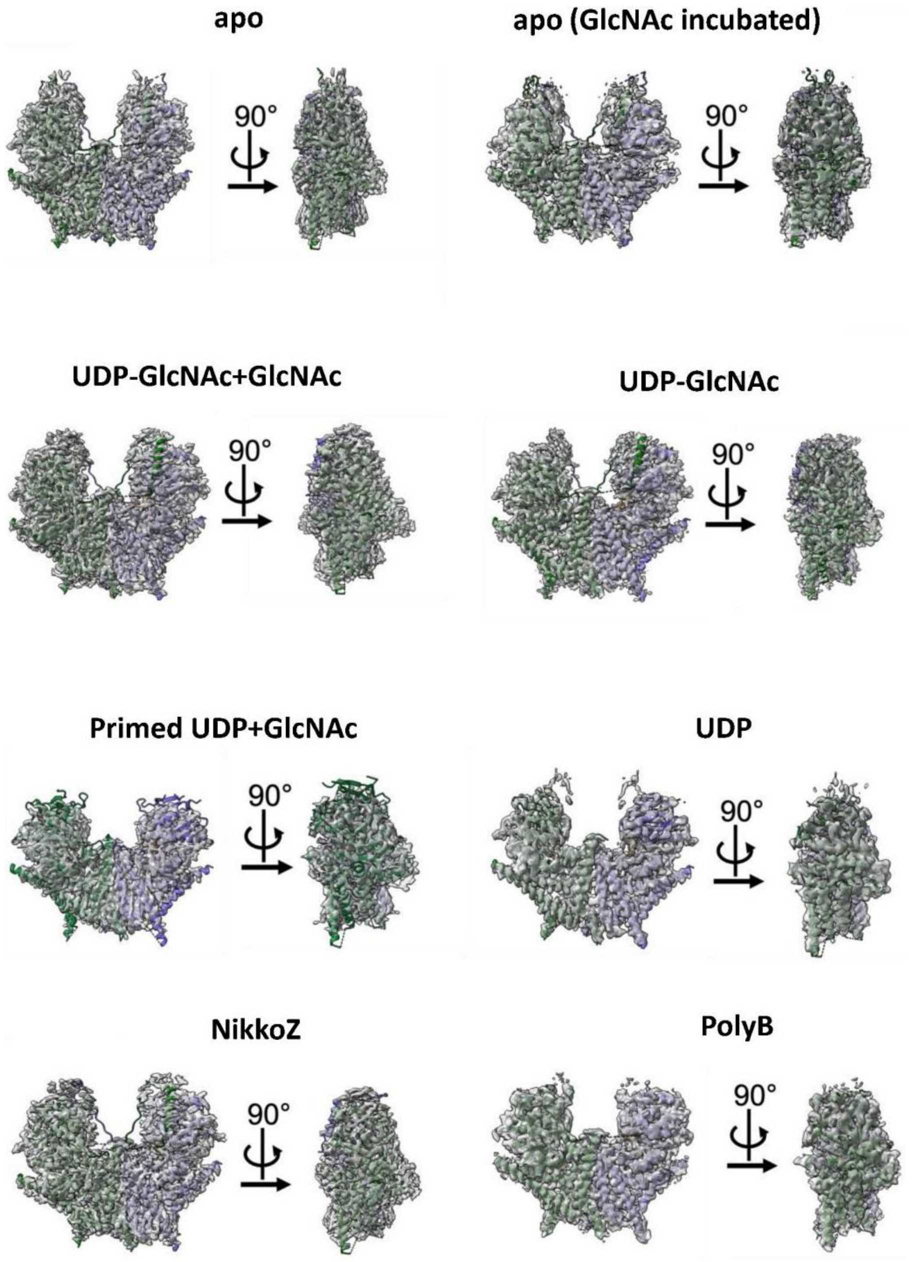

Supplementary Figure 12. Cryo-EM maps of Chs1 in apo, apo (GlcNAc incubated), UDP-GlcNAc+GlcNAc bound, UDP-GlcNAc bound, primed UDP+GlcNAc bound, UDP bound, NikkoZ bound, and PolyB bound states superimposed with their corresponding models.

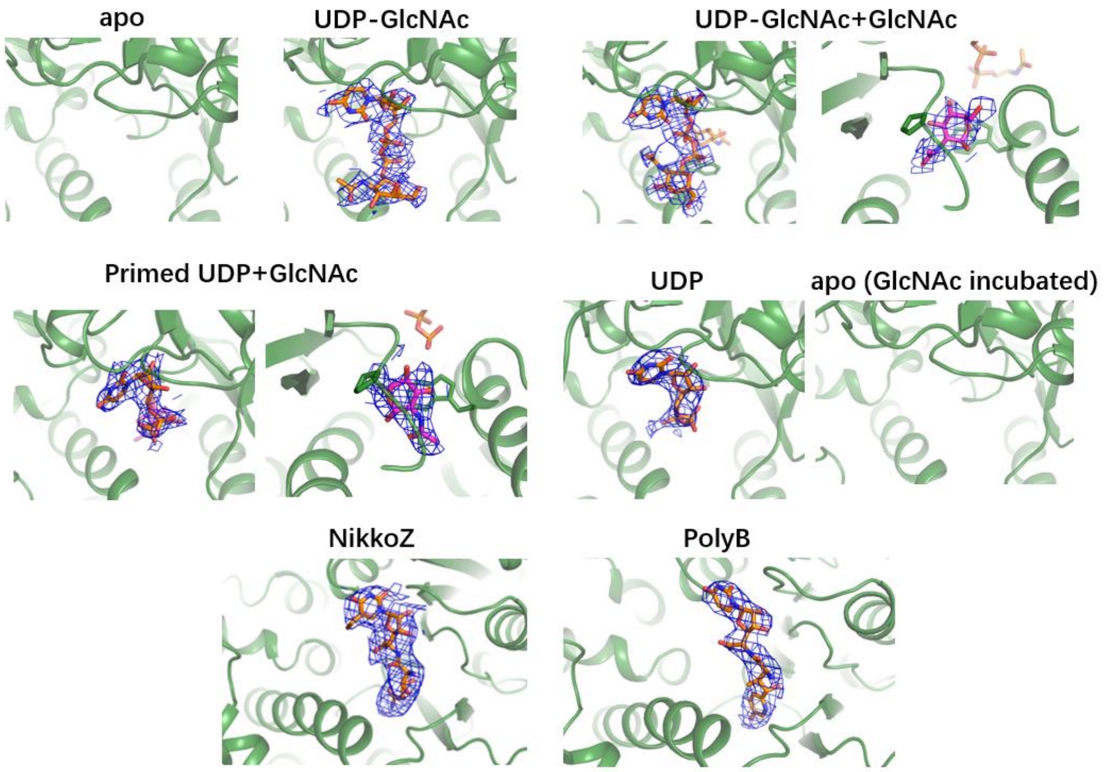

Supplementary Figure 13. Cryo-EM densities of substrates in structures of Chs1 in apo, UDP-GlcNAc bound, UDP+GlcNAc bound (primed), UDP-GlcNAc+GlcNAc bound, UDP bound, apo (GlcNAc incubated), NikkoZ bound, and PolyB bound states. The structure of Chs1 is shown as a green cartoon. UDP-GlcNAc, UDP, and inhibitors are shown as orange sticks. The GlcNAc acceptor is shown as purple sticks. Mg^{2+} molecules are in lime spheres.

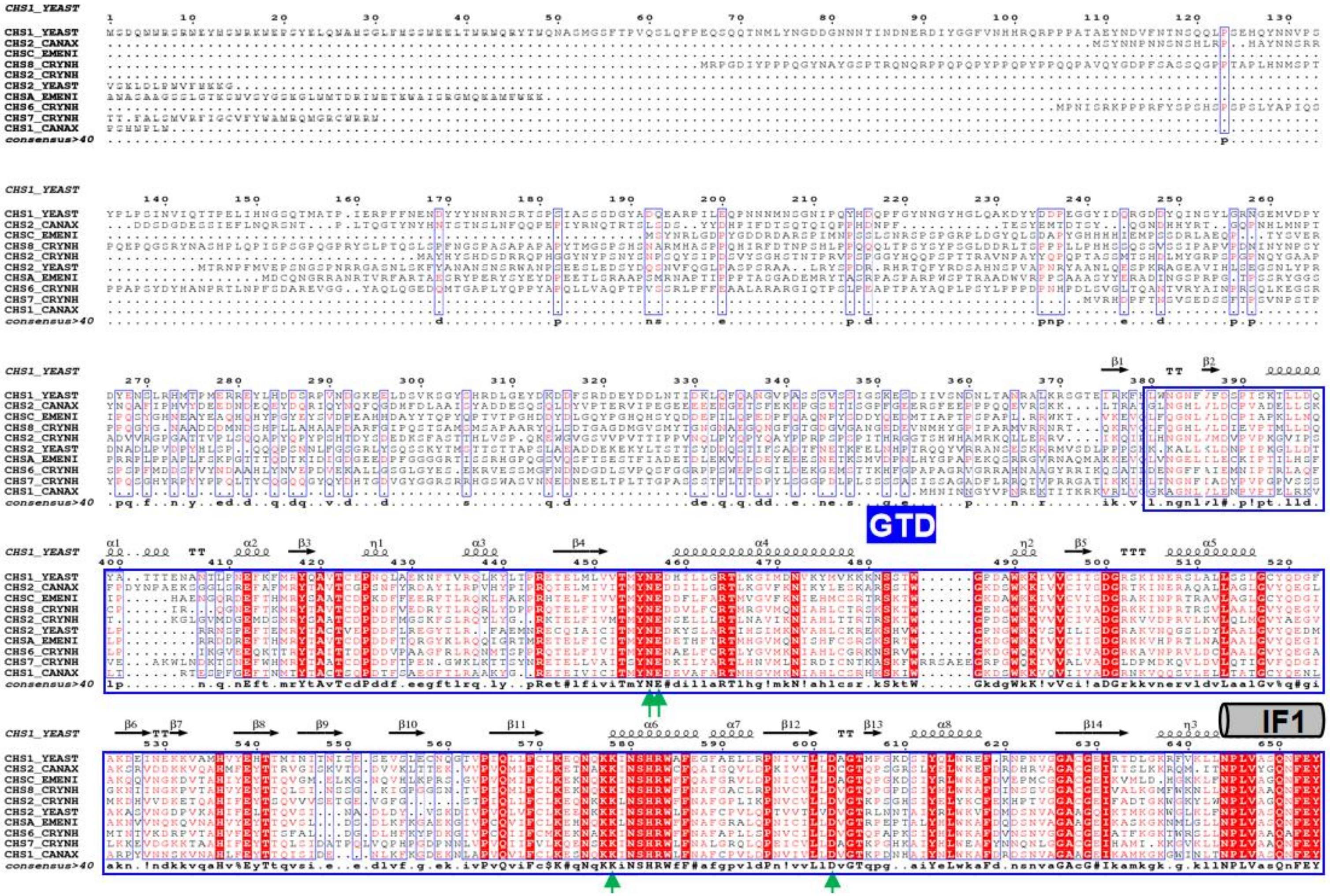
CHS1_YEAST
CHS2_CANAX
CHSC_EMENI
CHS8_CRYNH
CHS2_CRYNH
CHS2_YEAST
CHSA_EMENI
CHS6_CRYNH
CHS7_CRYNH
CHS1_CANAX
consensus>40
GTD
IF1

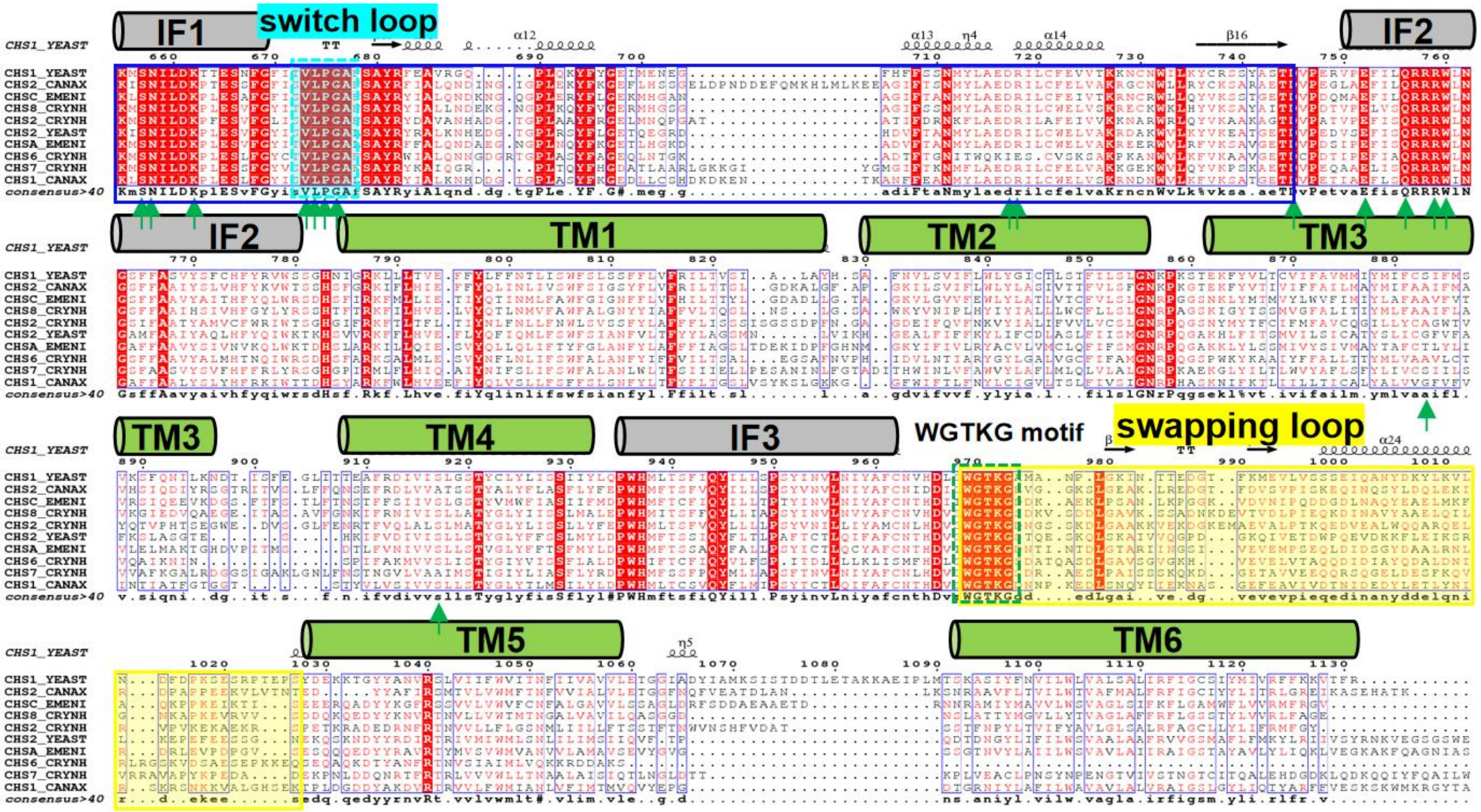

Supplementary Figure 14. Sequence alignment of chitin synthase genes from different fungal species. YEAST: *Saccharomyces cerevisiae*, CANAX: *Candida albicans,* EMENI: *Emericella nidulans,* CRYNH: *Cryptococcus neoformans*. GTD is highlighted by a blue rectangle. Transmembrane helices are highlighted by green cylinders according to our Chs1 structure. Interface helices (IF1–3) are highlighted by black or gray cylinders. The switch loop, domain swapping loop, and WGTKG motif are highlighted by cyan, yellow, and green rectangles respectively. Green arrows indicate key residues in the active site and transporting channel.

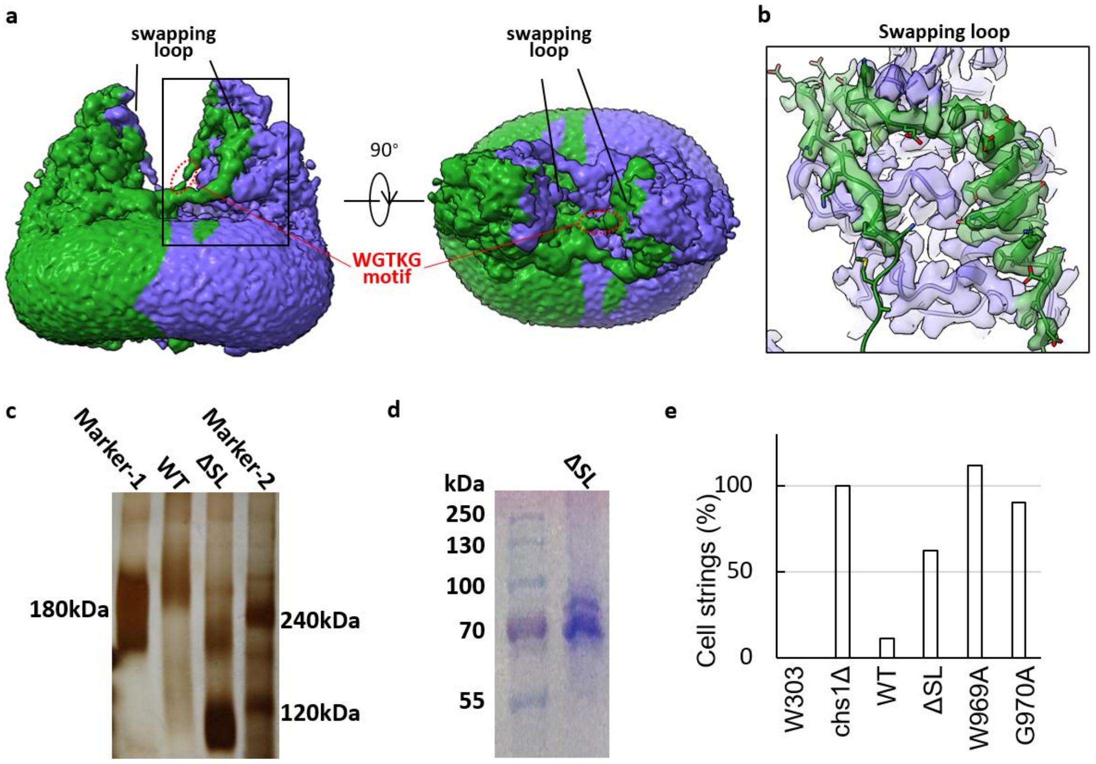

Supplementary Figure 15. Characterization of the swapping loop. a) Unsharped map of apo Chs1 is shown at a lower display threshold to show the weaker density. Location of WGTKG motif was highlighted by red ovals. Location of the swapping loops are labeled. The swapping loop of one subunit was highlighted by a black rectangle. **b**) An enlarged view of the region in the red box in (a). **c**) Purified swapping loop truncated Chs1 (Δ SL) was checked by native–PAGE gel with wild type Chs1 (WT) and two markers as references. Source data is provided as a Source Data file. Three independent experiments were conducted with similar results. **d**) Purified ΔSL was checked by Coomassie blue-stained SDS–PAGE gel. Source data is provided as a Source Data file. Three independent experiments were conducted with similar results. **e**) Growth complementation of wild-type w303 cells (w303) and chs1Δ cells with empty plasmid (chs1 Δ) or plasmid carrying either wild-type Chs1 (WT) or mutants (Δ SL, W969A and G970A). Representative cell images are shown in **Supplementary Fig. 15**. Cell string refers to a string of unseparated cells with number ⩾3. For each of these strains, >2000 cells were counted and calculated using w303 and chs1Δ as negative and positive controls. The phenotype of the cell string is an indicator of how much Chs1's function is disrupted in vivo.

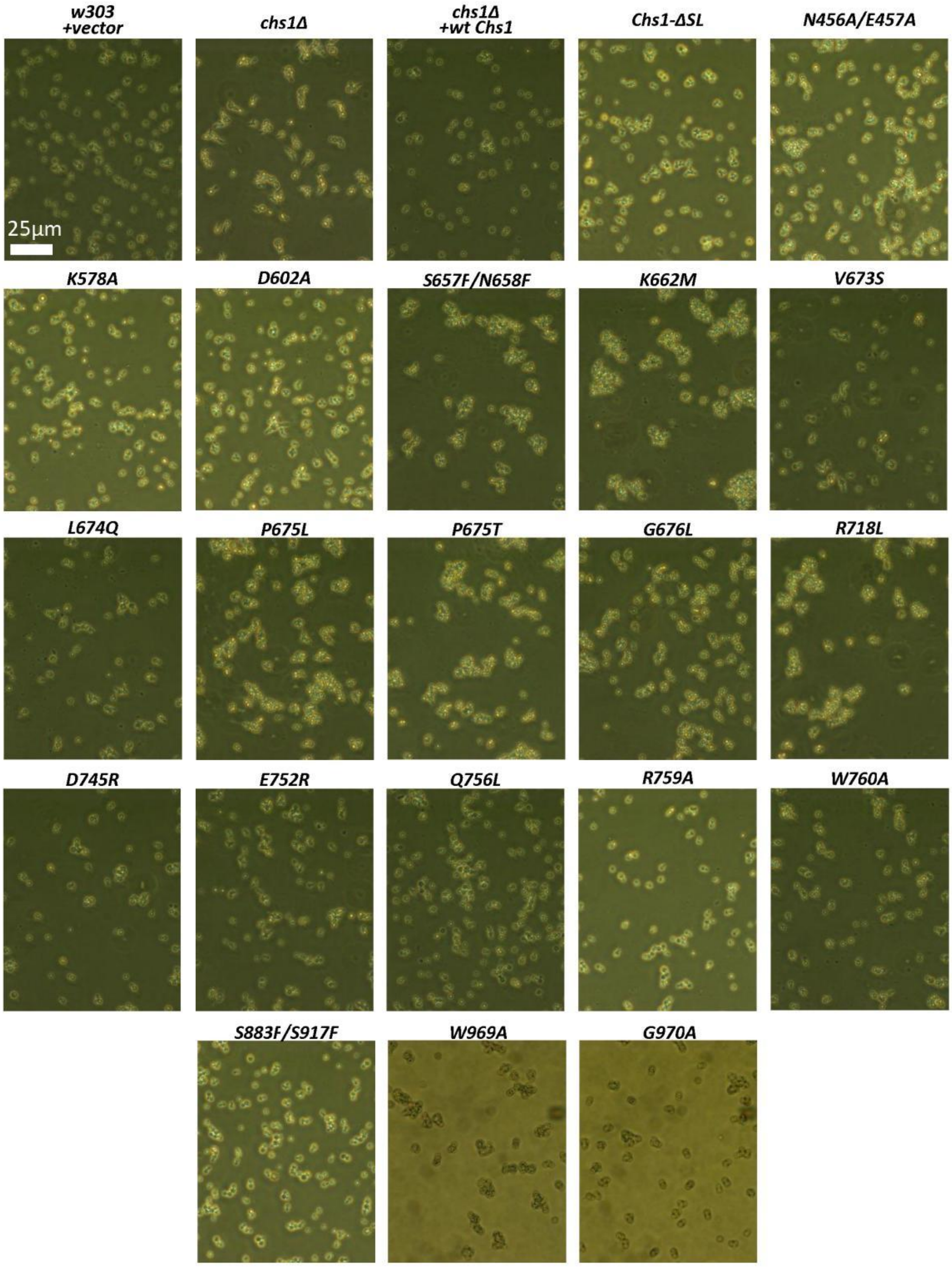

Supplementary Figure 16. Growth complementation of wild-type w303 cells with empty plasmid (w303+vector) and *chs1*Δ cells with empty plasmid (*chs1*Δ) or plasmid carrying either wild-type Chs1 (*chs1*Δ+wt Chs1) or mutants. Cell images were captured when cells grew to OD=1. The phenotype of the cell string is an indicator of how much Chs1's function is disrupted in vivo. Three independent experiments were conducted with similar results.

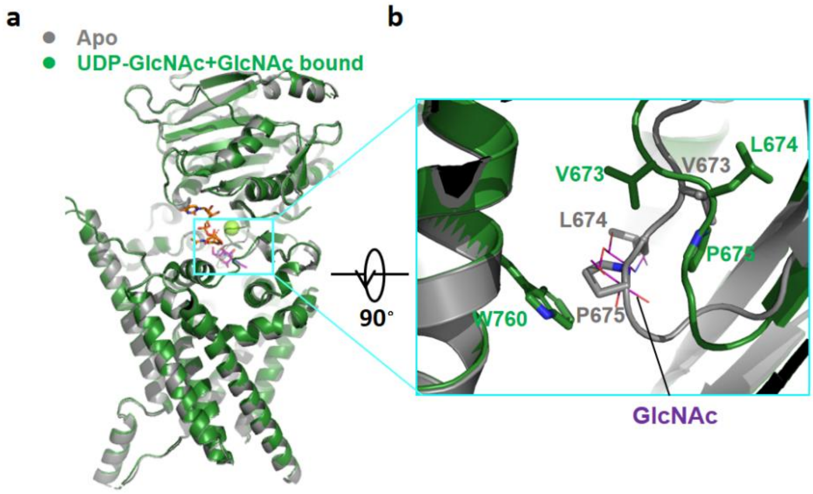

Supplementary Figure 17. Structural superposition between Chs1 in the apo state (gray) and UDP-GlcNAc+GlcNAc-bound state (green). a) Overall structure of apo Chs1 and UDP-GlcNAc+GlcNAc bound Chs1 in cartoon. The active site is highlighted by a cyan box. **b**) Enlargement of the cyan box region in (**a**) viewed from cytosolic side. GlcNAc acceptor (purple) is shown in line. W760 and residues of switch loop are shown in stick. Side chains of V673, L674, and P675 in two states face opposite orientations.

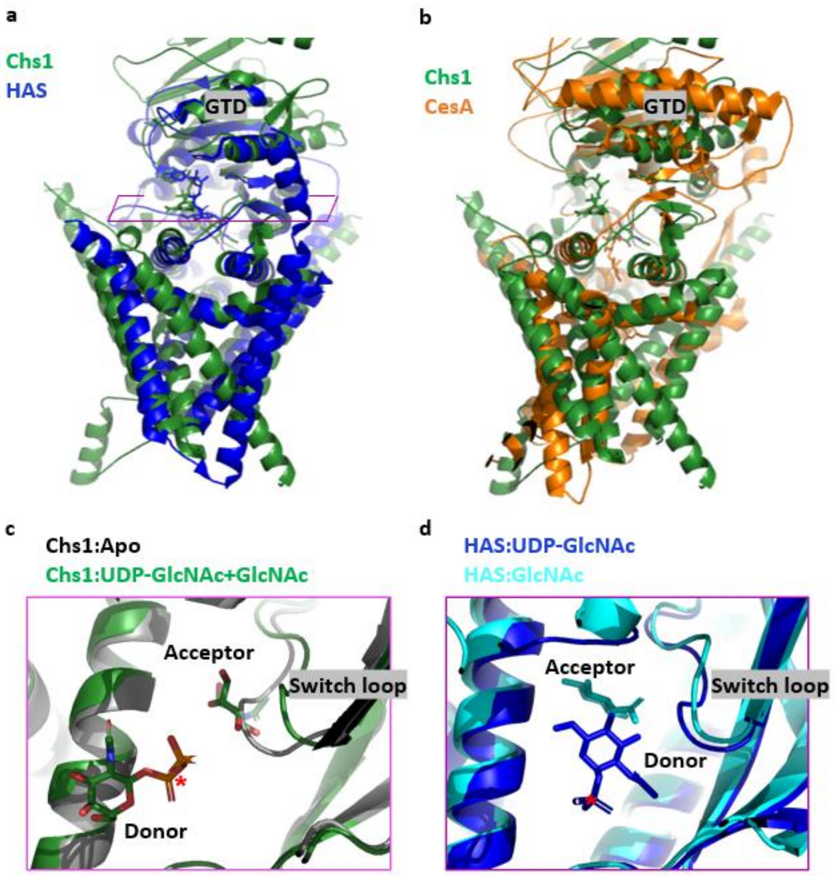

Supplementary Figure 18. Structural comparison between Chs1 and a hyaluronan synthase (HAS) and a cellulose synthase (CesA) by aligning their respective TMs. **a**) Structural comparison between UDP-GlcNAc+GlcNAc bound Chs1 (green) and UDP-GlcNAc bound HAS (PDB ID: 7SP8, blue) by aligning their respective TMs. The purple rhomboid mark the position for cut-in view shown in panels **c** and **d**. **b**) Structural comparison between UDP-GlcNAc+GlcNAc bound Chs1 (green) and cellulose bound CesA (PDB ID: 7D5K, orange) by aligning their respective TMs. **c**) A cut-in view from the cytosolic side of the superposition of Chs1 in the apo state (gray) and UDP-GlcNAc+GlcNAc bound state (green). The switch loop is shifted by ~4 Å. The GlcNAc moiety of UDP-GlcNAc faces outside, and the β-phosphate is highlighted by a red star. **d**) A cut-in view from the cytosolic side of the superposition of HAS in the UDP-GlcNAc bound state (blue) and GlcNAc bound state (cyan). The switch loop only shifted slightly upon UDP-GlcNAc hydrolysis in HAS. The GlcNAc moiety of UDP-GlcNAc in the HAS structure faces inside, and the β-phosphate is highlighted by a red star.

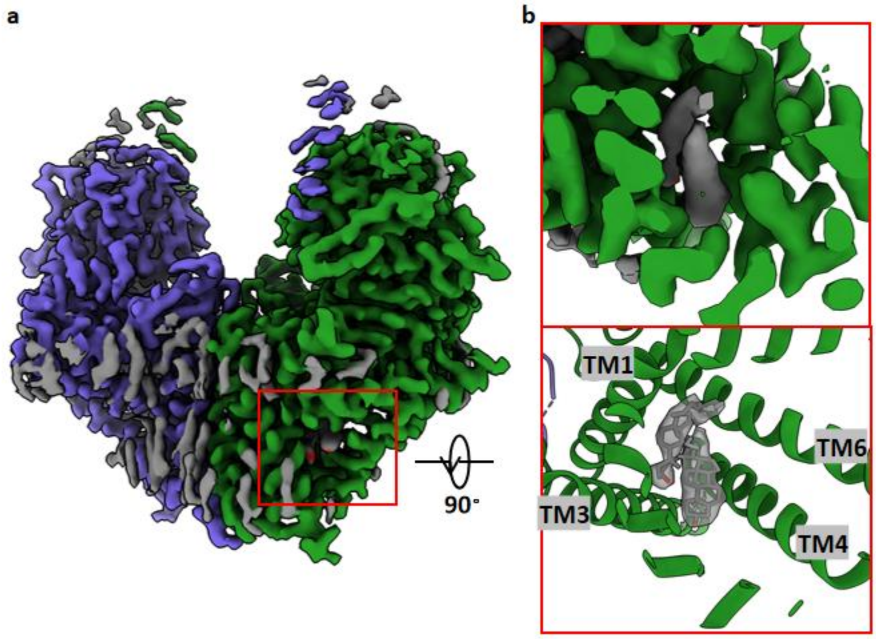

Supplementary Figure 19. Lateral opening of Chs1 is occupied by two sterol molecules. a) Overall map of Chs1. The lateral opening in the membrane region is highlighted by a red box. **b**) An enlarged view of the region in the red box in panel A rotated by 90°.

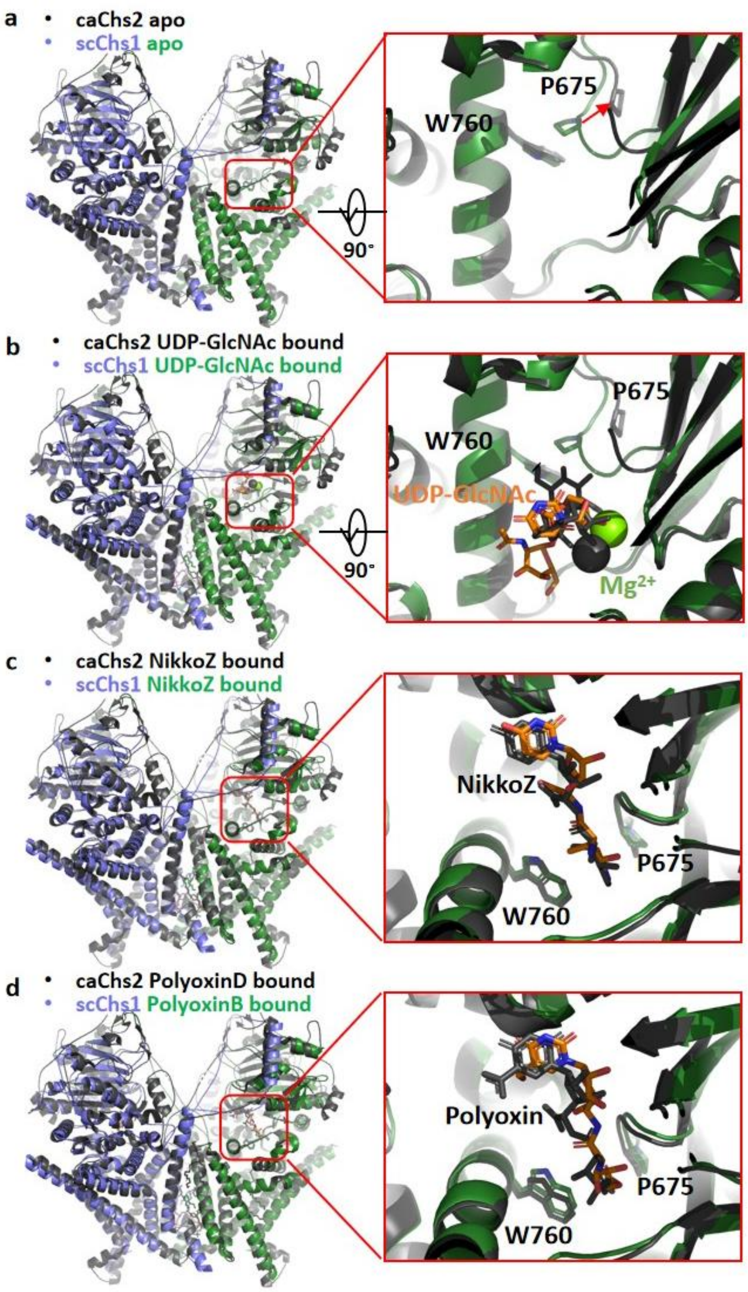

Supplementary Figure 20. Structural comparison between Chs1 (slate and green) and Candida albicans Chs2 (black) in apo (a)(PDB ID: 7STL), UDP-GlcNAc bound (b)(PDB ID: 7STM), NikkoZ bound (c)(PDB ID: 7STN), and Polyoxin bound (d)(PDB ID: 7STO) states. The active sites are highlighted by red boxes. Zoomed-in view of active sites are shown in right panel. P675 of the switch loop and W760 of IF2 are labeled. The position of P675 indicates the switch loop is closed in apo and UDP-GlcNAc bound Chs1, but opened in apo and UDP-GlcNAc bound caChs2.
