# Supplementary material for: Structure, catalysis, chitin transport, and selective inhibition of chitin synthase
Source: Nat Commun. 2023 Aug 8;14:4776. doi: 10.1038/s41467-023-40479-4 (PMC10409773; doi:10.1038/s41467-023-40479-4)
Supplement: Supplementary file 3 — Description of Additional Supplementary Files [file 41467_2023_40479_MOESM3_ESM.pdf]

Description of Additional Supplementary Files

File Name: Supplementary Movie 1

Description: 3DVA analysis of the donor-bound Chs1 structure, revealing the flexibility of the domain swapping loop and TMH4.
