# Supplementary material for: Structure, catalysis, chitin transport, and selective inhibition of chitin synthase
Source: Nat Commun. 2023 Aug 8;14:4776. doi: 10.1038/s41467-023-40479-4 (PMC10409773; doi:10.1038/s41467-023-40479-4)
Supplement: Supplementary file 5 — Reporting Summary [file 41467_2023_40479_MOESM5_ESM.pdf]

nature portfolio

Corresponding author(s): Cai-Hong Yun, Lin Bai

Last updated by author(s): Jul 18, 2023

Reporting Summary

Nature Portfolio wishes to improve the reproducibility of the work that we publish. This form provides structure for consistency and transparency in reporting. For further information on Nature Portfolio policies, see our Editorial Policies and the Editorial Policy Checklist.

Statistics

For all statistical analyses, confirm that the following items are present in the figure legend, table legend, main text, or Methods section.

n/a	Confirmed	
☐	☒	The exact sample size (n) for each experimental group/condition, given as a discrete number and unit of measurement
☐	☒	A statement on whether measurements were taken from distinct samples or whether the same sample was measured repeatedly
☒	☐	The statistical test(s) used AND whether they are one- or two-sided<br>*Only common tests should be described solely by name; describe more complex techniques in the Methods section.*
☒	☐	A description of all covariates tested
☒	☐	A description of any assumptions or corrections, such as tests of normality and adjustment for multiple comparisons
☐	☒	A full description of the statistical parameters including central tendency (e.g. means) or other basic estimates (e.g. regression coefficient) AND variation (e.g. standard deviation) or associated estimates of uncertainty (e.g. confidence intervals)
☒	☐	For null hypothesis testing, the test statistic (e.g. F, t, r) with confidence intervals, effect sizes, degrees of freedom and P value noted<br>*Give P values as exact values whenever suitable.*
☒	☐	For Bayesian analysis, information on the choice of priors and Markov chain Monte Carlo settings
☒	☐	For hierarchical and complex designs, identification of the appropriate level for tests and full reporting of outcomes
☒	☐	Estimates of effect sizes (e.g. Cohen's d, Pearson's r), indicating how they were calculated

Our web collection on statistics for biologists contains articles on many of the points above.

Software and code

Policy information about availability of computer code

Data collection	Cryo-EM data were collected automatically with SerialEM.
Data analysis	RELION-3.1, MotionCorr2-1.4.O, CTFFIND4.1, cryoSPARC-3.1, ChimeraX 1.2.5, Pymol (2.06), Coot (version 0.9.5), Phenix (version 1.19.2-4158-000), and MolProbity (version 4.5).

For manuscripts utilizing custom algorithms or software that are central to the research but not yet described in published literature, software must be made available to editors and reviewers. We strongly encourage code deposition in a community repository (e.g. GitHub). See the Nature Portfolio guidelines for submitting code & software for further information.

Data

Policy information about availability of data

All manuscripts must include a data availability statement. This statement should provide the following information, where applicable:

- Accession codes, unique identifiers, or web links for publicly available datasets
- A description of any restrictions on data availability
- For clinical datasets or third party data, please ensure that the statement adheres to our policy

The cryo-EM 3D maps and the corresponding atomic models of Chs1 have been deposited in the EMDB database and the RCSB PDB with the respective accession codes EMD-36856 [https://www.ebi.ac.uk/pdbe/entry/emdb/EMD-36856] and 8K3Q [http://doi.org/10.2210/pdb8K3Q/pdb] (apo state), EMD-36857 [https://www.ebi.ac.uk/pdbe/entry/emdb/EMD-36857] and 8K3R [http://doi.org/10.2210/pdb8K3R/pdb] (GlcNAc incubated apo state), EMD-36863 [https://www.ebi.ac.uk/

pdbe/entry/emdb/EMD-36863] and 8K3W [http://doi.org/10.2210/pdb8K3W/pdb] (UDP-GlcNAc+GlcNAc bound state), EMD-36862 [https://www.ebi.ac.uk/pdbe/entry/emdb/EMD-36862] and 8K3V [http://doi.org/10.2210/pdb8K3V/pdb] (UDP-GlcNAc bound state), EMD-36861 [https://www.ebi.ac.uk/pdbe/entry/emdb/EMD-36861] and 8K3U [http://doi.org/10.2210/pdb8K3Updb] (primed UDP+GlcNAc bound state), EMD-36859 [https://www.ebi.ac.uk/pdbe/entry/emdb/EMD-36859] and 8K3T [http://doi.org/10.2210/pdb8K3T/pdb] (UDP bound state), EMD-36855 [https://www.ebi.ac.uk/pdbe/entry/emdb/EMD-36855] and 8K3P [http://doi.org/10.2210/pdb8K3P/pdb] (PolyB bound state), and EMD-36864 [https://www.ebi.ac.uk/pdbe/entry/emdb/EMD-36864] and 8K3X [http://doi.org/10.2210/pdb8K3X/pdb] (NikkoZ bound state).
Other models used for analysis in this paper can be accessed in RCSB PDB database with their respective accession codes: UDP-GlcNAc bound HAS (PDB ID: 7SP8 [http://doi.org/10.2210/pdb7SP8/pdb]), CesA (PDB ID: 7D5K [http://doi.org/10.2210/pdb7D5K/pdb]), and Candida albicans Chs2 in apo (PDB ID: 7STL [http://doi.org/10.2210/pdb7STL/pdb]), UDP-GlcNAc bound (PDB ID: 7STM [http://doi.org/10.2210/pdb7STM/pdb]), NikkoZ bound (PDB ID: 7STN [http://doi.org/10.2210/pdb7STN/pdb]), and Polyoxin bound (PDB ID: 7STO [http://doi.org/10.2210/pdb7STO/pdb]) states.

Research involving human participants, their data, or biological material

Policy information about studies with human participants or human data. See also policy information about sex, gender (identity/presentation), and sexual orientation and race, ethnicity and racism.

Reporting on sex and gender	N/A
Reporting on race, ethnicity, or other socially relevant groupings	N/A
Population characteristics	N/A
Recruitment	N/A
Ethics oversight	N/A

Note that full information on the approval of the study protocol must also be provided in the manuscript.

Field-specific reporting

Please select the one below that is the best fit for your research. If you are not sure, read the appropriate sections before making your selection.

☒ Life sciences ☐ Behavioural & social sciences ☐ Ecological, evolutionary & environmental sciences

For a reference copy of the document with all sections, see nature.com/documents/nr-reporting-summary-flat.pdf

Life sciences study design

All studies must disclose on these points even when the disclosure is negative.

Sample size	We collected eight cryo-EM datasets: 1)1914 micrographs with Chs1 in apo state; 2)1564 micrographs with Chs1 in apo state after incubation with GlcNAc; 3)3045 micrographs with Chs1 in UDP-GlcNAc bound state; 4) 2469 micrographs with Chs1 in UDP+GlcNAc bound state; 5) 1356 micrographs with Chs1 in UDP-GlcNAc+GlcNAc bound state; 6) 632 micrographs with Chs1 in UDP bound state; 7) 2311 micrographs with Chs1 in polyoxin B bound state; 5) 2711 micrographs with Chs1 in nikkomycin Z bound state.The sample size was not predetermined. The sample size was deemed appropriate when it led to 3D maps at desired resolution. For functional assays, each reaction was performed 3 times.
Data exclusions	"Bad" raw particles that did not produce 2D class averages or 3D class maps with defined features were excluded after 2D and 3D classifications. This criteria is empirical but is a standard image processing practice in the cryoEM community.
Replication	Reproducibility resides in the large number of particles used to derive at the final 3D maps or 2D averages. The reliability and the resolution is measured by gold-standard Fourier shell correlation. Replication efforts with multiple refinement runs (n>3) yielded was successful, yielding similar 3D maps. For functional assays, all reaction were repeatable.
Randomization	The raw particles were automatically selected by computer program (RELIONS 3.0). Randomization is not relevant to this study.
Blinding	The investigators were not blinded to the specific data points during data collection and analysis of cryo-EM analysis. There is no need for blinding in this type of study. For functional assays, the investigators were blinded during signal measurement and analysis.

Reporting for specific materials, systems and methods

We require information from authors about some types of materials, experimental systems and methods used in many studies. Here, indicate whether each material, system or method listed is relevant to your study. If you are not sure if a list item applies to your research, read the appropriate section before selecting a response.

Materials & experimental systems

n/a	Involved in the study	
☐	☒	Antibodies
☐	☒	Eukaryotic cell lines
☒	☐	Palaeontology and archaeology
☒	☐	Animals and other organisms
☒	☐	Clinical data
☒	☐	Dual use research of concern
☒	☐	Plants

Methods

n/a	Involved in the study	
☒	☐	ChIP-seq
☒	☐	Flow cytometry
☒	☐	MRI-based neuroimaging

Antibodies

Antibodies used

Anti-Flag mouse monoclonal antibody (Abcam, Catalog: ab125243, Clone: FG4R)

Validation

Product name: Anti-DDDDK tag (Binds to FLAG® tag sequence) antibody [FG4R]
Description: Mouse monoclonal [FG4R] to DDDDK tag (Binds to FLAG® tag sequence)
Host species: Mouse
Specificity: Recognizes the N-terminal, C-terminal or internal DYKDDDDK-tagged (also known as the FLAG® tag sequence) fusion proteins.
Tested applications Suitable for: WB, ELISA, Dot blot, IP, In situ hybridization

Eukaryotic cell lines

Policy information about cell lines and Sex and Gender in Research

Cell line source(s)

S. cerevisiae strain BY4742 purchased from Horizon Discovery,Dharmacon

Authentication

N/A

Mycoplasma contamination

N/A

Commonly misidentified lines
(See ICLAC register)

N/A
