# Supplementary material for: Structure, catalysis, chitin transport, and selective inhibition of chitin synthase
Source: Nat Commun. 2023 Aug 8;14:4776. doi: 10.1038/s41467-023-40479-4 (PMC10409773; doi:10.1038/s41467-023-40479-4)
Supplement: Supplementary file 6 — Source Data [file 41467_2023_40479_MOESM6_ESM.zip › Source_data1_gel_rawimages.docx]

Source Data file for

**Structure, catalysis, chitin transport, and selective inhibition of chitin synthase**

Dan-Dan Chen, Zhao-Bin Wang, Le-Xuan Wang, Peng Zhao, Cai-Hong Yun, and Lin Bai

This file contains the original images used in Supplementary Figure 1b, 1c, 1d, 3a, 3b, 14c, 14d

**Supplemental Figure 1b**


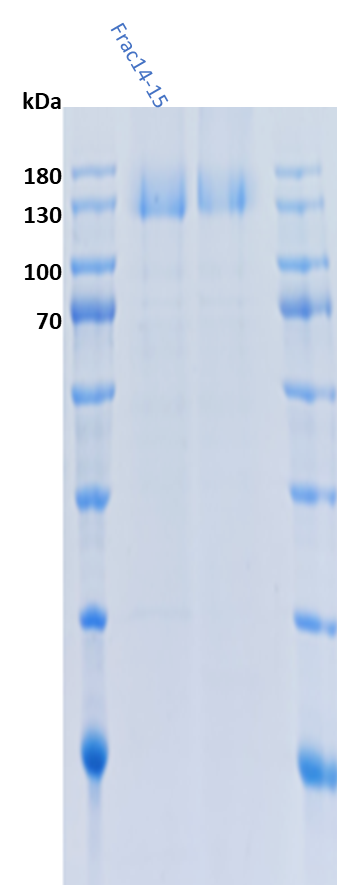


**Supplemental Figure 1c**


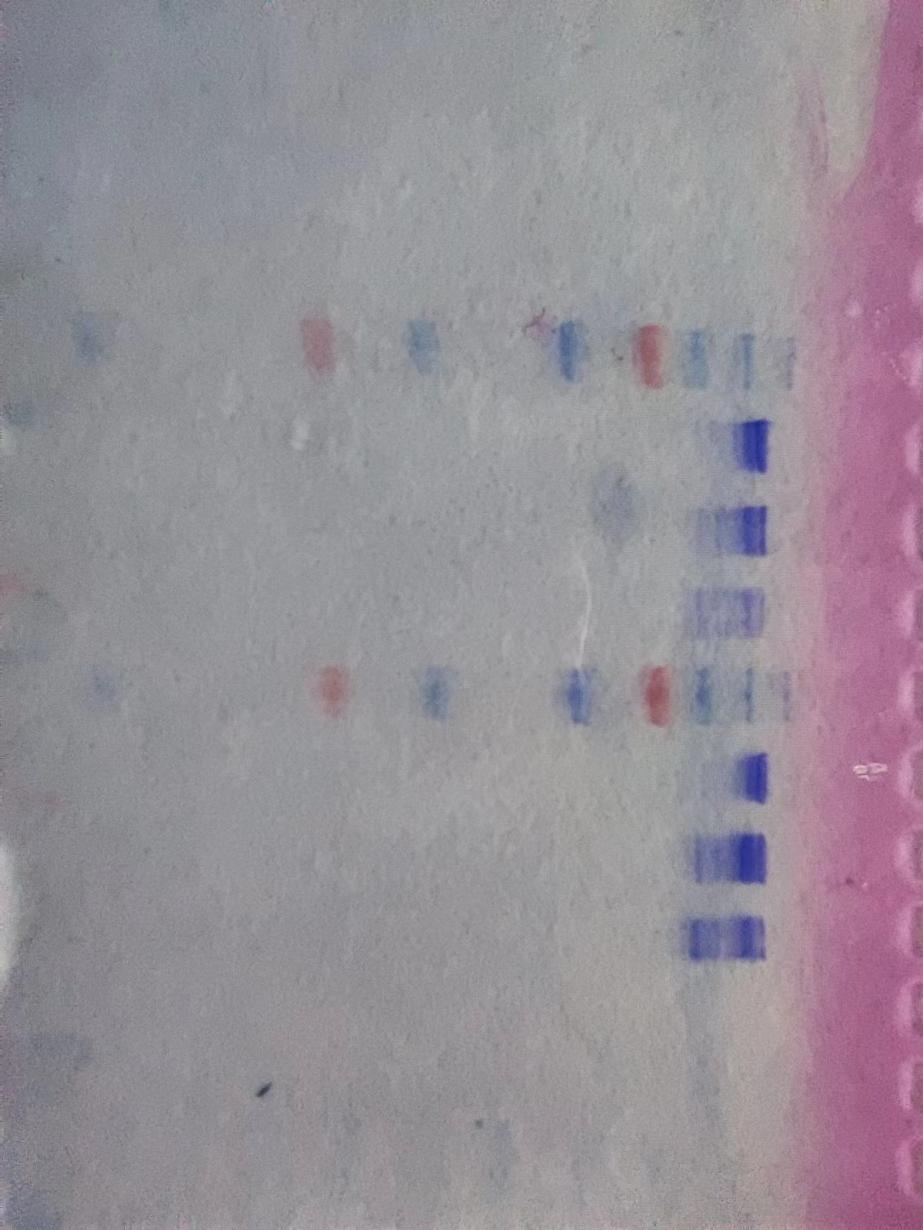


**Other samples**

**Frac14-15**

**Frac13-14**

**kDa**

**250**

**130**

**100**

**70**

**Supplemental Figure 1d**

**Membrane**

**Frac13-14**

**Other samples**

**kDa**

**250**

**130**

**100**

**70**


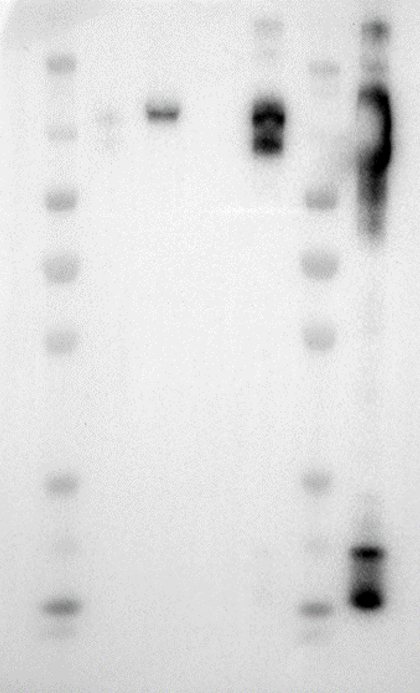


**Supplemental Figure 3a**

| **Chs1:Trypsin** | | | | | | |
| --- | --- | --- | --- | --- | --- | --- |
| **8k** | **8k** | **4k** | **4k** | **2k** | **1k** | **500** |
| **5min** | **15** | **5** | **15** | **15** | **15** | **15** |

**ΔN+Trypsin**

**Chs1**

**kDa**

**250**

**130**

**100**

**70**

**55**

**Other samples**

**ΔN**


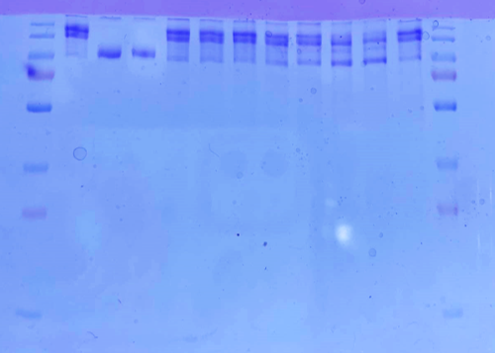


**Supplemental Figure 3b**


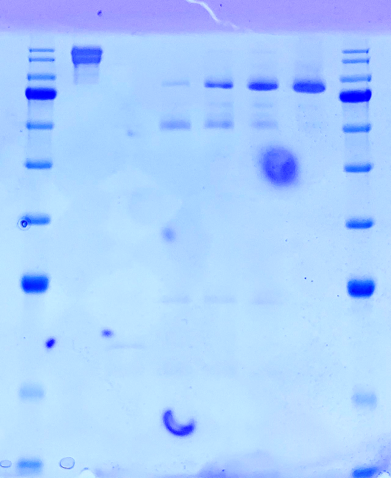


**Chs1**

| **Chs1:Trypsin=500** | | | | |
| --- | --- | --- | --- | --- |
| **10h** | **5h** | **2h** | **1h** | **30min** |

**kDa**

**180**

**130**

**100**

**70**

**Supplemental Figure 14c**


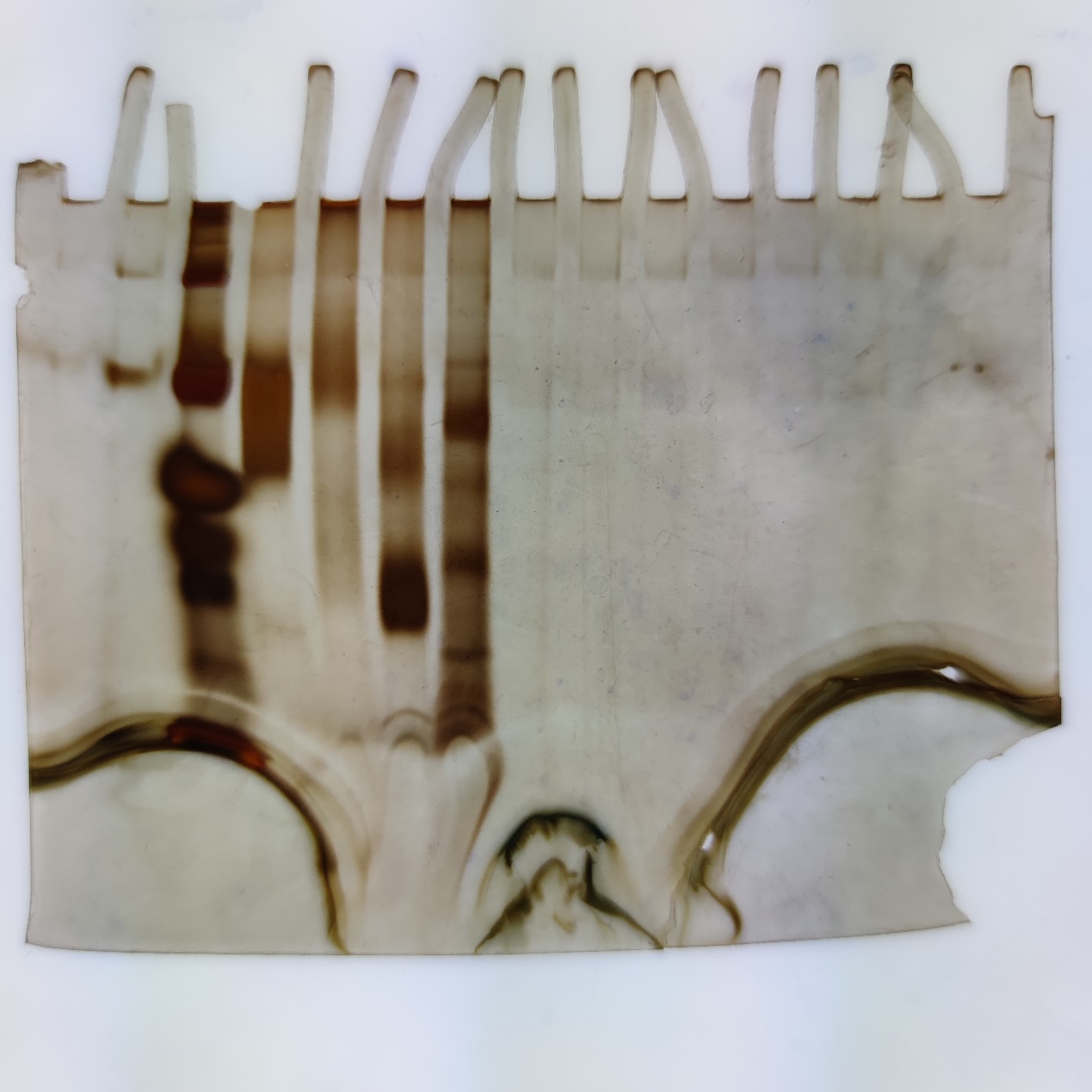


**Other samples**

**Marker-1**

**120kDa**

**240kDa**

**180kDa**

**ΔSL**

**WT**

**Marker-2**

**Supplemental Figure 14d**


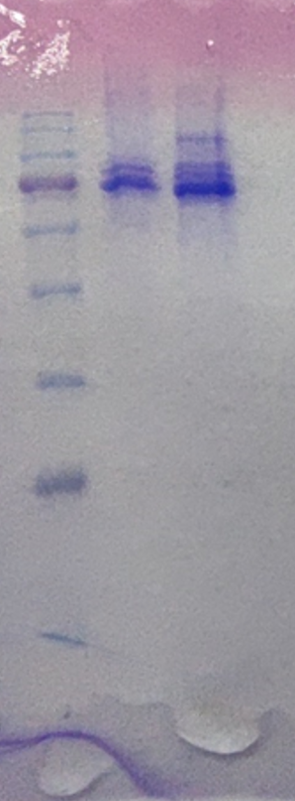


**kDa**

**250**

**130**

**100**

**70**

**55**

**Other sample**

**ΔSL**
